# Multiple intrinsic and extrinsic drivers influence the quantity and quality components of seed dispersal effectiveness in the rare shrub *Lindera subcoriacea*

**DOI:** 10.1371/journal.pone.0283810

**Published:** 2023-03-31

**Authors:** Matthew G. Hohmann, Wade A. Wall, Michael G. Just, Stacy D. Huskins

**Affiliations:** 1 US Army Corps of Engineers, Engineer Research and Development Center, Champaign, Illinois, United States of America; 2 Endangered Species Branch, Fort Bragg, North Carolina, United States of America; Southeastern Louisiana University, UNITED STATES

## Abstract

Information about seed dispersal effectiveness (SDE) for plant species of conservation concern is rarely available to inform management strategies and actions. For *Lindera subcoriacea* (bog spicebush, Lauraceae), a rare endemic dioecious shrub of the southeastern United States, we examined the influence of two intrinsic and five extrinsic drivers on the number and proportion of seeds either dispersed, or predated pre- and post-dispersal. The number of seeds dispersed characterizes the quantitative component of SDE, while pre- and post-dispersal seed predation can affect the qualitative component of SDE. Using fruit counts, seed traps, and seed removal depots over multiple years, we estimated that approximately 28% of *L*. *subcoriacea* seeds are lost to pre-dispersal predation, 69% of seeds are dispersed, 3% of seeds fail to disperse, and 65% of dispersed seeds are predated post-dispersal. We observed substantial variation in these three processes among individuals. We also found that both intrinsic (plant height, crop size) and extrinsic (understory cover, time since last fire, conspecific fruiting neighborhood, substrate) drivers differentially influenced the three processes. We identified four generalist, seasonally frugivorous, avian visitors at fruiting individuals that likely act as variably effective dispersers, while the Northern Cardinal (*Cardinalis cardinalis* L.) is a seed predator. Rodent granivores were important pre- and post-dispersal seed predators. The magnitude of our pre-dispersal and post-dispersal seed predation estimates suggest that, given the low fecundity of *L*. *subcoriacea*, conservation strategies should emphasize facilitating dispersal and reducing the effects of seed predation.

## Introduction

Seed dispersal plays a central role in plant reproduction, with large impacts on individual fitness, population demography, metapopulation dynamics, and genetic structure [1–3]. Seed dispersal is affected by multiple interacting processes across multiple stages and can be challenging to study. Seed dispersal effectiveness (SDE) has become a widely adopted conceptual framework for evaluating endozoochorous dispersal of fleshy-fruited species [3, 4], and can be applied to emphasize the SDE that dispersers provide or that plants receive. Seed dispersal effectiveness is calculated as the product of quantitative and qualitative components. The quantitative component of SDE characterizes the number of seeds dispersed and is commonly estimated based on two subcomponents, the number of visits and the number of seeds dispersed per visit. Typically, an assemblage of generalist disperser species contribute to the overall quantitative component of SDE [e.g., 5, 6]. The qualitative component of SDE characterizes the probability a dispersed seed produces a new adult, although seedling establishment is a commonly evaluated proxy for species with long maturation times. The qualitative component is also comprised of two subcomponents, quality of treatment and quality of deposition. Quality of treatment characterizes how the handling of seeds by dispersers (e.g., in the mouth or gut) affects seed survival and can be either facilitative or antagonistic. For example, seed germinability may increase after removal of fruit pulp [7, 8], while seed breakage and digestion—seed predation—typically reduce seed survival [3, 9]. Quality of deposition characterizes the various abiotic and biotic factors that affect seed and seedling fate post-dispersal, including germination and edaphic conditions, as well as exposure to predators, pathogens, herbivores, competitors, and disturbance regimes [10].

Numerous spatially and temporally dynamic intrinsic (i.e. variation in traits of individual plants) and extrinsic (i.e. variation in ecological context) drivers are expected to generate intraspecific variation in SDE [11]. In their recent review, Schupp et al. [12] found that fruit crop size and fruit/seed size are the most studied and understood intrinsic drivers, whereas plant height is not well-studied. For crop size, there is a consistent positive relationship between the number of seeds dispersed, but not the proportion of seeds dispersed. In contrast, fruit/seed size has been shown to have both positive and negative effects on the quantity component of SDE [12], likely due to the differential ability of different sized dispersers to accommodate variation in fruit/seed size. For example, the bill gape width of avian dispersers is generally a limiting factor for the consumption of different sized fruits [13]. Plant height also likely has variable effects on dispersal, as vertical segregation of frugivores with different dispersal traits can lead to variation in SDE among individual plants of different heights [e.g., 14].

For extrinsic drivers, Schupp et al. [12] found that local environmental conditions and habitat structure are the primary drivers of SDE variation, whereas fruiting neighborhood likely plays a lesser role. A diversity of habitat-related variables have been shown to influence the quantity and quality components of SDE at ecological scales ranging from microhabitats to landscapes and include among others community composition, cover density, and vertical structure, as well as habitat fragmentation, disturbance, and degradation [15–17]. The magnitude and direction of these extrinsic drivers vary across study systems likely due to inter and intraspecific variability in disperser traits and behaviors such as body size, age, sex, food preferences, digestive efficiencies, etc. [e.g., 18–20]. Similarly, fruiting neighborhood can either negatively influence the quantity component of SDE by generating inter and intraspecific competition for dispersers [e.g., 21–23], or positively influence dispersal quantity through facilitation [e.g., 24, 25]. With regard to the quality component of SDE, the density of fruiting neighborhoods has generally been shown to reduce dispersal distances [e.g., 26,27].

For rare species with limited reproductive output, the importance of SDE is amplified due to the greater consequence of any single seed on individual fecundity and population demography [28]. Therefore, information about SDE and how the individual components and subcomponents are affected by intrinsic and extrinsic drivers is of particular interest for rare plant species and potentially valuable for developing effective conservation strategies. For example, if the quantity of dispersed seeds is low within small populations of a rare plant species due to small conspecific fruiting neighborhoods [12], enhancing the population size and/or density via seedling propagation and outplanting could help to promote SDE quantity. If quality of deposition is low due to small seed shadows and negative conspecific density dependence [29], transporting and planting seeds into suitable sites may enhance recruitment and population growth rates [30].

*Lindera subcoriacea* Wofford (bog spicebush) is a rare (i.e. small range, habitat specialist, sparse local abundance; [31]) dioecious shrub of the southeastern USA that has experienced a 28% population decline across a substantial portion of its range during the last 30 years [32]. Although not previously studied, the red, fleshy, lipid-rich, single-seeded drupes of *L*. *subcoriacea* are likely consumed by birds [33, 34] and the presumably short lifespan of dispersed seeds (1–2 years) precludes any development of a seedbank [35, 36]. *Lindera subcoriacea* has limited and highly skewed fruit production [37], with most females producing few or no fruits annually and a few individuals producing a relatively large number. When deposited in suitable habitats, *L*. *subcoriacea* has an average combined germination and seedling survival rate of 7% one year post-dispersal, and < 1% are recruited (i.e. surviving two years post-dispersal) [37].

The rarity, small size, and declining number of *L*. *subcoriacea* populations [32], as well as limited information about the species, suggest an improved understanding of SDE would be invaluable for conservation planning and proactive management. In this paper, we examine multiple processes related to *L*. *subcoriacea* SDE (Table 1). Specifically, we explore how the number and proportion of seeds dispersed (quantitative component proxy), predated pre-dispersal (qualitative treatment subcomponent proxy), and predated post-dispersal (qualitative deposition subcomponent proxy) are affected by four extrinsic drivers known to influence foraging behavior and efficiency: vegetation community occupied, understory cover, time since last fire, and conspecific fruiting neighborhood size [e.g., 38–40]. For the number and proportion of seeds dispersed and predated pre-dispersal, we also assess the importance of two intrinsic drivers, individual fruit crop size and plant height. For post-dispersal predation, we additionally assess the influence of local substrate composition [e.g., 41]. Both the number and proportion of seeds removed have been examined in previous SDE studies [e.g., 12], with the number characterizing quantitative effectiveness and the proportion characterizing efficiency [e.g., 42,43]. By assessing the magnitude and variation in these three processes, we seek to expand what is known about the seed ecology of this rare species and provide conservation recommendations.

**Table 1:** Intrinsic and extrinsic drivers investigated for effects on the quantitative component and qualitative subcomponents of seed dispersal effectiveness in *L*. *subcoriacea*.

Driver type	Driver	Quantitative (dispersal)	Qualitative (predation)	
			Treatment (pre-dispersal)	Deposition (post-dispersal)
Intrinsic	Individual crop size	X	X	
	Height	x	X	
Extrinsic	Fruiting neighborhood	X	X	
	Vegetation community	X	X	X
	Understory cover	X	X	X
	Time since last fire	X	X	X
	Substrate characteristics			X

## Methods

### Ethics statement

No permits were required of our research team to access sites on the publicly-owned, federally-managed property where the study occurred, as the study was collaboratively conducted with the land manager. Field studies did not involve any state- or federally-protected species.

### Study system and species

U.S. Army Garrison Fort Bragg (35°8’21"N, 78°59’57"W) spans approximately 73,468 ha in the Sand Hills ecoregion (hereafter Sandhills) of south-central North Carolina [44]. The Sandhills are a matrix of xeric uplands and mesic/hydric lowlands (i.e. wetlands). The xeric uplands have a savanna physiognomy that is maintained by fire and dominated by a longleaf pine (*Pinus palustris* Mill.) canopy [45]. Growing season (April–September) prescribed fires are scheduled on a 3-year rotation to approximate the mean historic fire return interval [46]. While the understory of the uplands typically burns during prescribed fires, fires penetrate wetlands much less frequently [37, 47, 48]. Fire kills aboveground herbaceous and shrub biomass and significantly reduces the size and fecundity of woody resprouts for the first several years post-fire [49–51].

The majority of *L*. *subcoriacea* populations within North Carolina are known from Fort Bragg and have been observed in four wetland vegetation communities [32, 47, 52]: Streamhead Pocosins, Sandhill Streamhead Swamps, Streamhead Atlantic White Cedar Forests, and Sandhill Seeps. The four communities differ in fire frequency, hydrology, canopy cover, understory cover, and other environmental factors [32]. Streamhead Pocosins have a relatively open canopy dominated by *Pinus serotina* Michaux and a dense understory dominated by a variety of evergreen shrub species (*Ilex coriacea* (Pursh) Chapman and *I*. *glabra* (L.) Gray are the dominant fleshy-fruited species). Sandhill Streamhead Swamps have a canopy composed of *Nyssa biflora* Walter, *Acer rubrum* L., and *Liriodendron tulipifera* L., with an understory of evergreen shrub species. In general, the canopy of Sandhill Streamhead Swamps is more closed and the understory less dense, relative to Streamhead Pocosins. Streamhead Atlantic White Cedar Forests have the highest canopy cover of the four vegetation communities, with the canopy containing at least 50% Atlantic white cedar (*Chamaecyparis thyoides* L.) coverage. Finally, Sandhill Seeps typically occur midslope where erosion has exposed the clay subsoils, leading to semipermanently saturated conditions at the surface. Sandhill Seeps are generally smaller in area, have a relatively open overstory and midstory, and support a larger herbaceous component relative to the other three vegetation communities. Sandhill Streamhead Swamps and Streamhead Atlantic White Cedar Forests have low relative fire frequency, Streamhead Pocosins have medium relative fire frequency, and Sandhill Seeps have high fire frequency [32].

The single-seeded drupes of *L*. *subcoriacea* mature simultaneously in late July and early August (S1 Fig), and although not documented in the species, are most likely avian-dispersed based on fruit traits [53–55] and observations for congeners [34, 56]. Although *L*. *subcoriacea* has fruits/seeds of similar size to *L*. *benzoin* (ovate seeds are 7.02 ± 0.29 mm, *n* = 101 [37] and 7.02 ± 0.11 mm, *n* = 50 [57] long, respectively), *L*. *subcoriacea* individuals produce fewer fruits than *L*. *benzoin* (80 ± 178, *n* = 290 [37] and 164 ± 104, *n* = 11 [58], respectively). Fruit production in both species likely varies as a function of individual size and habitat conditions that affect productivity (e.g., light, water and nutrient availability) [59], but for *L*. *subcoriacea* habitat effects are obscured by high variability in individual fruit production [37].

### Potential seed dispersers and predators

In a separate effort, we developed post-breeding avian occupancy models for Fort Bragg based on survey data collected during mid-August 2017 and 2018 [60] and coinciding with our seed dispersal and predation studies (next section). Occupancy models accounted for imperfect detection [61], as implemented in the R (R Development Core Team, 2021) package *unmarked* using the double-observer approach [62]. We documented 22 seasonally frugivorous species (as described by numerous publications; e.g.,[33, 58]) during the survey, and generated spatial occupancy estimates at a 30 x 30 m resolution for 20 of these species. Many of these species are expected to consume *L*. *subcoriacea* fruits and disperse seeds, given that bill gape widths exceed the size of *L*. *subcoriacea* fruits/seeds. One exception is the Northern Cardinal (*Cardinalis cardinalis* L.), which is primarily granivorous during the non-breeding season [63]. For five avian species documented visiting fruiting *L*. *subcoriacea* (see next section), we compared potential differences in the magnitude of their roles as seed dispersers or predators by calculating their mean occupancy at 88 georeferenced female plants across 69 *L*. *subcoriacea* populations [32, 37]. We assessed differences in these mean occupancy estimates among species pairs with bootstrapped confidence intervals. In addition to the Northern Cardinal, as many as ten different rodent species are likely to predate *L*. *subcoriacea* seeds in our study system post-dispersal [e.g., 64, 65].

### Estimating seed dispersal and pre-dispersal predation with fruit counts and seed traps

Over three years (2017–2019), we used seed traps to estimate the number and proportion of *L*. *subcoriacea* fruits that were dispersed, fell from the maternal plant, or predated pre-dispersal. We constructed seed traps having two 1 x 1 x 0.08 m wooden interlocking frames, elevated on four 1 m wooden legs. We covered the upper frame with galvanized hardware cloth (1.26 cm^2^ mesh) to allow fallen fruits to pass and be intercepted by stainless steel mesh window screen covering the lower frame. This design ensured that fallen fruits could not be removed by animals or precipitation. We placed a single seed trap beneath 26 of 88 (29.5%) annually monitored female individuals [37]. We chose individuals and subjectively positioned traps based on the efficacy of trap placement, which was influenced by shrub branching structure (horizontal reach ≥ 1 m), approximately level topography, and number of fruits. Traps encompassed approximately 15–25% of the individual canopies. We positioned traps in mid-July of each year, before *L*. *subcoriacea* fruits begin to ripen (S1 Fig). While deploying the traps, we counted the number of ripe and unripe fruits on each individual (107.7 ± 231.9; 12–1500) and the subset directly above the trap (57.9 ± 98.6; 4–624) that would be intercepted if they were to fall from the plant instead of being removed by a disperser or seed predator. We revisited individuals every 3–4 days to 1) recount the remaining ripe and unripe fruits on individuals and above the seed traps; 2) recover and count whole fallen fruits, regurgitated whole seeds, and remnants of predated fruits and seeds intercepted by the traps; and 3) record fecal evidence of rodent visitors. We revisited seed traps for 30 days, or until no fruits remained on the shrub, whichever came first.

For each individual plant (*n* = 26; 42 total observations across 3 years), we estimated 1) dispersal as the number and proportion of fruits counted above the seed trap that were removed, but neither recovered as whole fruits, regurgitated whole seeds, nor as fruit/seed fragments in the trap; 2) pre-dispersal predation as the number and proportion of fruits counted above the seed trap that were found as fruit/seed fragments in the trap; and 3) dispersal failure as the number and proportion of fruits counted above the seed trap that were found as whole fruits, or regurgitated whole seeds within the trap. We acknowledge that predation of fruits/seeds counted above seed traps could have taken place elsewhere, resulting in underestimation. Additionally, it is possible that fruits/seeds removed from neighboring plants may have been deposited in the seed traps beneath our focal plants, which would cause pre-dispersal predation and dispersal failure to be overestimated.

Each year we also placed ≥ 1 Reconyx HyperFire PC800 infrared camera (Holmen, WI, USA) at a subset of the *L*. *subcoriacea* individuals where seed traps were positioned and at several additional fruiting individuals without seed traps to collect observations of diurnal and nocturnal endothermic visitors that may function as seed predators or dispersers. The dense vegetation within communities occupied by *L*. *subcoriacea*, particularly Streamhead Pocosins where most populations occur, severely limits observations of frugivores and seed predators. At distances ≥ 2 m, it typically becomes impossible to clearly see individual *L*. *subcoriacea*. Therefore, we chose individuals based on our ability to position the camera(s) to have an unobstructed line(s) of sight on fruit-bearing branches. We mounted cameras at 1.5 m above the ground on tripods located ≤ 2 m from target individuals, set them on motion detection photographic mode, and left them in place for the entire period during which fruit counts were collected and seed traps were monitored. Cameras were positioned for a total of 264,000 (25 cameras on 20 individuals), 63,360 (12 cameras on 10 individuals), and 25,344 hrs (8 cameras on 6 individuals), in 2017, 2018, and 2019, respectively. We visually reviewed images recorded by the cameras for the presence of seed predators and frugivores, documenting species identity when possible. Observations were only used to identify visitors at fruiting individuals and not used in any quantitative analyses.

### Estimating post-dispersal seed predation with seed depots

In 2017 and 2019, we conducted 10-day seed removal experiments from August 1–14, coinciding with *L*. *subcoriacea* seed dispersal (S1 Fig). Using ArcGIS (ArcPro v. 2.2, ESRI, Redlands, CA, USA), we generated 5,000 random points within Streamhead Pocosins, Sandhill Streamhead Swamps, and Sandhill Seeps vegetation communities. Streamhead Atlantic White Cedar Forests were not included due to their rarity and limited distribution on Fort Bragg. We delineated the locations of the vegetation communities using the best available information, which was a map of historical (before 1750) vegetation [66]. Intensive use of prescribed fire and hardwood suppression on Fort Bragg during the past 30 years has resulted in a landscape that more closely resembles the pyroclimax vegetation states characterized in this historical map than the 20th century fire suppressed state. We organized a subset of the random points, which were separated by > 1 km, into routes (two in 2017; four in 2019) composed of approximately 15 points each that could be visited within a single day. Routes were located in the western and northwestern portion of the installation, where most *L*. *subcoriacea* populations occur. Although the location of route points was approximated by the location of the random points, the actual location was haphazardly chosen at the time of depot deployment to match one of the three vegetation communities. Points were located 20–50 m from the firebreaks (single lane trails) used to traverse the routes.

We installed a pair of seed removal depots separated by 1 m at 21 and 60 points during mid-July 2017 and 2019, respectively. We used two types of depots: 1) closed, allowing invertebrate seed predators access; and 2) open, allowing both invertebrate and small mammalian seed predators access. We constructed depots from inverted translucent plastic buckets (12.7 cm in height and 21.9 cm in diameter) with two 8 cm x 15 cm openings cut into opposite sides. We covered the openings of closed depots with galvanized hardware cloth (1.26 cm^2^ mesh) and left the openings of open depots unobstructed. We affixed mesh window screen to the inverted opening of both depot types to contain seeds, while preventing rainfall accumulation. Similarly designed seed depots have been successfully used in other seed predation studies conducted in longleaf pine ecosystems [e.g., 64, 67]. We deployed pairs of empty open and closed depots at points 10–14 days prior to seed presentation. On 4 successive days we travelled each of the routes and loaded depots at each point with 10 *L*. *benzoin* seeds that had been purchased from Sheffield’s Seed Company (Locke, NY, USA), vacuum sealed in a plastic bag, and heated to 100°C in a water bath for 10 minutes to kill the embryos. We used the seeds of *L*. *benzoin* due to a lack of *L*. *subcoriacea* seeds and assumed that the seed removal observed for this surrogate congener would be the same as for *L*. *subcoriacea*. The two species were only recently taxonomically separated and have similar sized seeds [68]. Each year several individual depots (6 in 2017; 11 in 2019) or depot pairs were compromised by fires, flooding, and animal damage, and removed from the dataset.

### Intrinsic and extrinsic drivers

We estimated individual height, individual crop size, and conspecific fruiting neighborhood using demographic data collected from females during 2017–2019 [37]. We calculated fruiting neighborhood by summing the individual crop sizes of all fruiting *L*. *subcoriacea* within 5, 10, and 30 m radius nested buffers of each seed trap. We did not include any other fruiting species occurring within buffers in the neighborhood estimates. Although the heterospecific fruiting neighborhood is also known have positive and negative effects on dispersal [23–25], difficulty moving through the dense shrubs in *L*. *subcoriacea* habitats precluded us from making accurate counts of fruits.

We estimated time since the last fire (TSLF) based on a previously-developed methodology for estimating fire occurrence [51]. Briefly, we used Landsat satellite imagery (30 x 30 m resolution) and several imagery indices, coupled with a Random Forest classifier, to identify areas that had burned on Fort Bragg. We classified all pixels from Landsat images from 1991–2019, as either burned, or not on an annual basis. We then estimated the mean TSLF within a 30 m radius buffer around each seed trap and depot location.

We estimated canopy and understory cover using Airborne Light Detection and Ranging (LiDAR) data acquired from several flights flown December 20–27, 2012. We first converted the LiDAR dataset into a 25 m^2^ raster image. We then summarized the number of aboveground (AG) and bare earth (BE) returns points within each cell and estimated canopy cover using the formula $canopy\;cover=\frac{AG}{AG+BE}$. We then classified the AG return points by height (< 0.25 m = ground, 0.25–5 m = low vegetation) and calculated understory cover using the formula $understory\;cover=\frac{low\;vegetation}{ground}$. Finally, we calculated the mean canopy and understory cover (proportion) within a 30 m radius of each seed trap and depot location. Although prescribed fires may have temporarily reduced understory cover during the 5–7 years between LiDAR data collection and our study, both the upland herbaceous vegetation (≤ 1 growing season) and the woody wetland vegetation (≥ 3 years) rapidly recovers to pre-burn size [49]. Across all three years of our study mean TSLF was ≥ 3 years for 90.5% of seed trap and 84% of depot locations. The majority of these seed trap (75%) and depot (59%) locations where TSLF was < 3 years were in Sandhill Seeps, which are generally small and isolated within the upland savanna matrix.

In 2019, we collected substrate cover data at each depot pair within two 1 m^2^ microplots centered on the depots and oriented to prevent spatial overlap. We collected data on the percentage cover of litter, fine woody debris (< 10 cm diameter), coarse woody debris (> 10 cm diameter), bare ground, herbaceous vegetation cover (< 1 m), and woody vegetation cover (< 1 m) observed in each microplot and recorded the mean of the paired plots.

### Data analyses

For our analyses, we used estimates of the number and proportion of seeds dispersed and predated that were based on the initial and final counts. We removed one individual with more than 400 fruits from the neighborhood estimates because of its outlier status. We explored the drivers for correlations and found minimal evidence of collinearity (|*r*| > 0.70 [69]; S1 and S2 Tables). We fit univariate, mixed-effects generalized linear regression models to investigate the effects of each intrinsic and extrinsic driver on the number and proportion of seeds dispersed and predated pre- and post-dispersal using the *lme4* package [70]. Seed trap and depot IDs were included as a random effect and habitat as a fixed effect. For proportion data, we used a binomial error structure with a logit link. For count data, we used a Poisson error structure with a log link with a random intercept. We included a quadratic term within models to account for possible non-linear effects of drivers. Full models included the driver and the quadratic term. We evaluated the effect of the potential drivers on model explanatory power by evaluating the full model against the reduced (without quadratic) model using a type II likelihood ratio χ^2^ test (base::anova; [71]) [72, 73]. If there was no difference in explanatory power between the full and reduced model, we did not include the quadratic term. As needed, we evaluated the explanatory power between the reduced model and null (intercept only) model using the same procedure. We performed all statistical analyses using the statistical platform R version 4.1 [74], with the script used to analyze the data available from the authors. Our threshold of statistical significance was α = 0.05. For all significant models, we calculated a pseudo R^2^ [75].

## Results

### Pre-dispersal seed predation and observed seed predators

**Fig 1:**
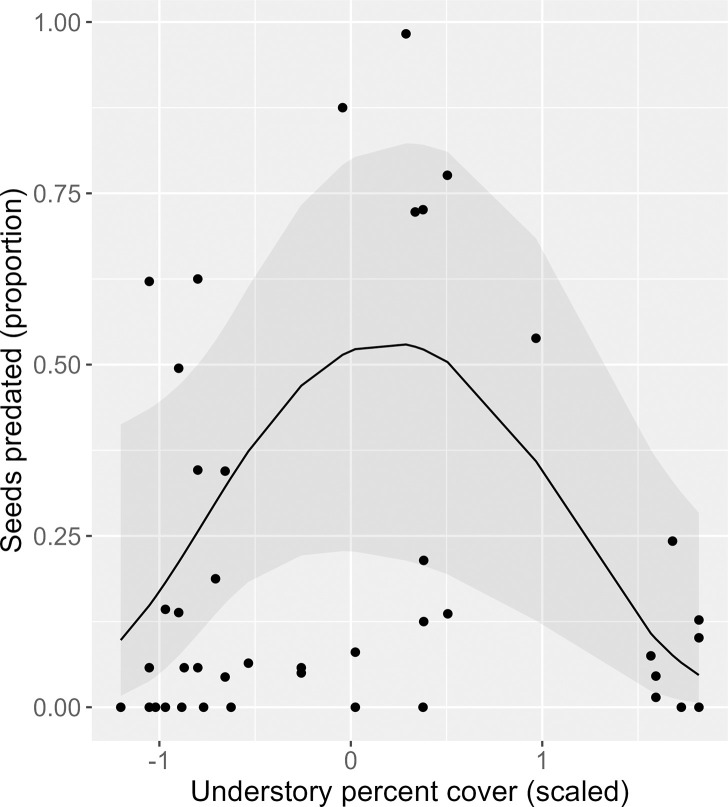
Relationship between pre-dispersal predation of *Lindera subcoriacea* seeds (proportion) and understory cover (scaled and centered). Points are individual observations, the line is from a mixed-effects generalized linear regression model with a quadratic effect (full model), and shaded region is the 95% confidence interval.

Across all years and individuals the mean proportion of pre-dispersal seed predation was 0.28 ± 0.06 SD. Most individuals experienced low pre-dispersal seed predation, but a few experienced high predation (range = 0.0–1.0) (S2 Fig). There was a negative quadratic effect of understory cover on the proportion of seeds predated ($\chi_{2\;df}^{2}$ = 4.79, *P* = 0.03), with individuals in low and high understory having relatively low seed predation compared to those in intermediate understory (Fig 1). No other intrinsic or extrinsic drivers had a significant effect on the number or proportion of seeds predated pre-dispersal (Table 2). Data and additional details about regression results can be found in S1 and S2 Files.

**Table 2:** Results testing for the significance of intrinsic and extrinsic drivers on treatment (pre-dispersal predation) and deposition (post-dispersal predation) subcomponents of seed dispersal effectiveness quality (number and proportion). Model comparisons evaluated with type II likelihood ratio χ^2^ tests are identified by letter codes after each driver for the number and proportion of seeds, respectively. *P* values in bold indicate a significant effect (α = 0.05).

Driver type	Driver (*SDE quality subcomponent*)	Number of seeds				Proportion of seeds			
		Pseudo R^2^	χ^2^	df	*P*	Pseudo R^2^	χ^2^	df	*P*
	***Treatment—pre-dispersal seed predation***								
Intrinsic	Individual crop size ^a^^,^^a^		11.3	1	0.59		1.31	1	0.25
	Height (scaled) ^a^^,^^a^		0.85	1	0.36		1.28	1	0.26
Extrinsic	Vegetation community ^a^^,^^a^		6.66	1	0.08		5.91	1	0.12
	Understory cover ^a^^,^^b^		0.12	1	0.72	0.02	4.79	2	**0.03**
	Time since last fire ^a^^,^^a^		1.3	1	0.24		0.78	1	0.38
	5 m fruiting neighborhood ^a^^,^^a^		0.86	1	0.72		0.20	1	0.66
	10 m fruiting neighborhood ^a^^,^^a^		1.83	1	0.18		0.01	1	0.91
	30 m fruiting neighborhood ^a^^,^^a^		4.97	1	0.08		3.00	1	0.22
	***Deposition—post-dispersal seed predation***								
Extrinsic	Vegetation community ^a^						4.11	1	0.13
	Understory cover ^a^						0.57	1	0.45
	Time since last fire ^a^						0.14	1	0.71
	Fine woody debris ^a^					0.02	3.91	1	**0.048**
	Coarse woody debris ^a^						1.19	1	0.28
	Litter ^a^						2.39	1	0.12
	Bare ground ^a^						2.94	1	0.09
	Woody cover ^a^						0.39	1	0.52
	Herbaceous cover ^a^						1.01	1	0.31

^a^Reduced (driver and random intercept) versus null (intercept only)

^b^Full (driver with a quadratic term and random intercept) versus reduced

Camera traps recorded one granivorous avian species at fruiting *L*. *subcoriacea*, the Northern Cardinal [63]. Within the grid cells occupied by 88 *L*. *subcoriacea* individuals across 69 populations on Fort Bragg the mean occupancy of the Northern Cardinal was 0.42 ± 0.11 [60]. Cameras also documented small nocturnal mammalian granivores likely in the genus *Peromyscus* within the canopies of fruiting *L*. *subcoriacea*. Fecal evidence of rodent visitors was observed in 45% of the seed traps where pre-dispersal predation was documented over the three years. In contrast, fecal evidence of rodent visitors was only observed in two seed traps where no pre-dispersal predation was documented.

### Seed dispersal and observed seed dispersers

**Fig 2:**
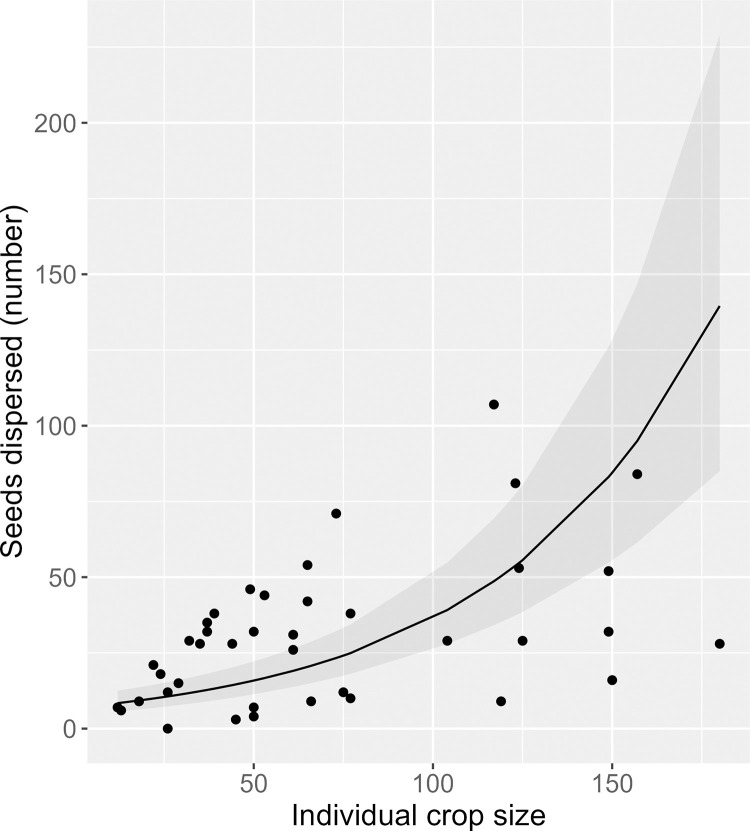
Relationship between the number of *Lindera subcoriacea* seeds dispersed and individual crop size. Points are individual observations, the line is from a mixed-effects generalized linear regression model (no quadratic term; reduced model), and the shaded region is the 95% confidence interval.

**Fig 3:**
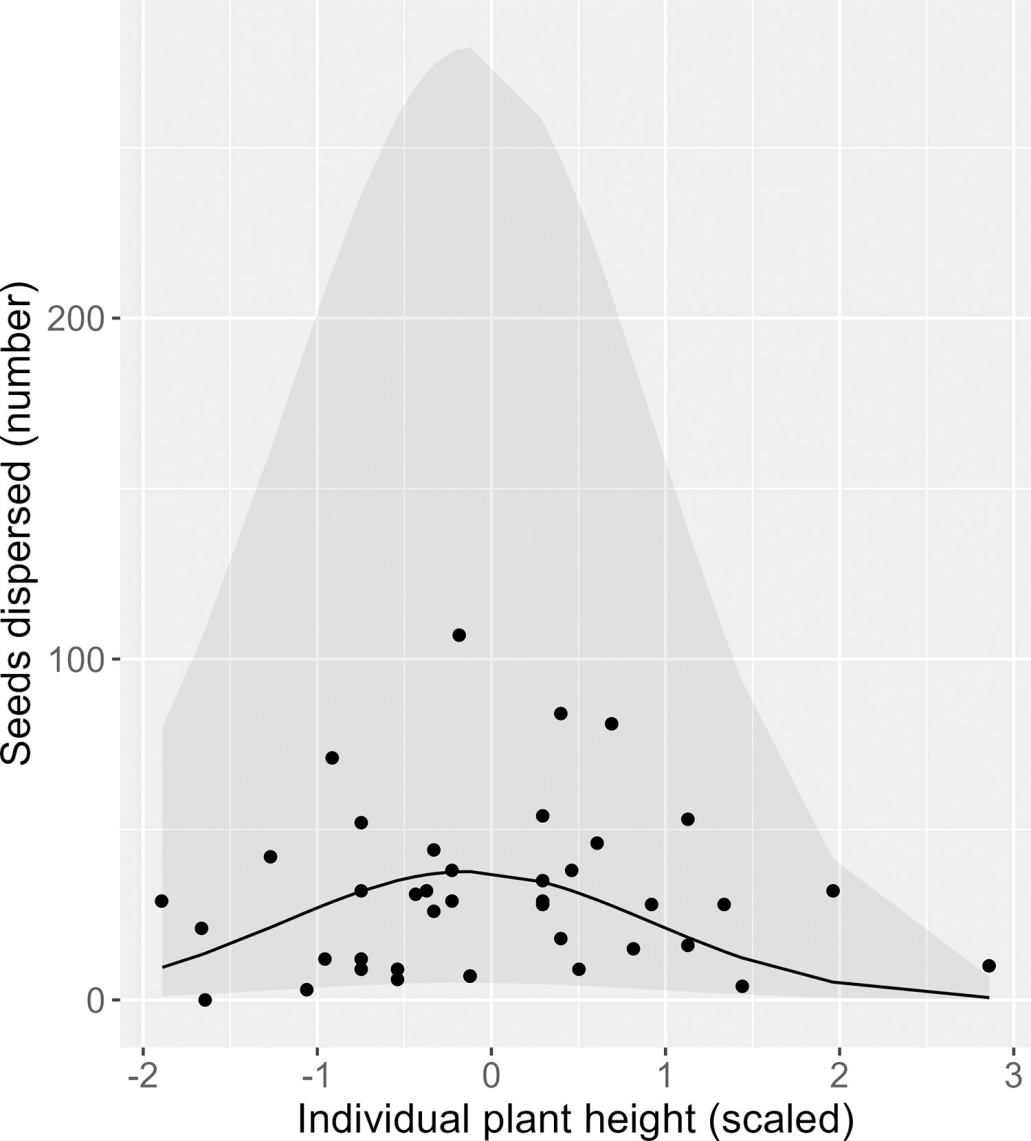
Relationship between the number of *Lindera subcoriacea* seeds dispersed and individual height (scaled). Points are individual observations, the line is from a mixed-effects generalized linear regression model with a quadratic effect (full model), and the shaded region is the 95% confidence interval.

**Fig 4:**
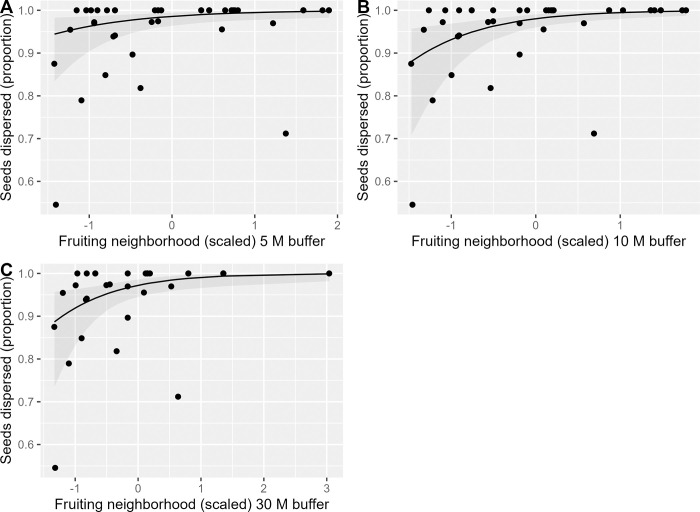
Relationship between proportion of *Lindera subcoriacea* seeds dispersed and fruiting neighborhoods (scaled and centered) within (A) 5 m, (B) 10 m, and (C) 30 m radius nested buffers. Points are individual observations, the lines are from mixed-effects generalized linear regression models (reduced models), and the shaded regions are 95% confidence intervals.

Across all years and individuals, the mean proportion of dispersed seeds was 0.69 ± 0.25. Of the seeds that were not predated pre-dispersal, a high mean proportion (0.96 ± 0.03) were estimated to be dispersed (S3 Fig), with the rest falling below maternal plants. A positive linear effect of individual crop size ($\chi_{1\;df}^{2}$ = 97.7, *P* < 0.001) and a negative quadratic effect of height ($\chi_{2\;df}^{2}$ = 32.2, *P* < 0.001) were documented for the number (Figs 2 and 3), but not the proportion of seeds dispersed (Table 3). There also was a positive linear effect of TSLF ($\chi_{1\;df}^{2}$ = 28.8, *P* < 0.001) on the number of seeds dispersed, and a positive quadratic effect of TSLF ($\chi_{2\;df}^{2}$ = 13.96, *P* < 0.001) on the proportion of seeds dispersed. In addition, there were positive linear effects of fruiting neighborhood on the number and proportion of seeds dispersed; effects were observed at the 5 and 10 m scales for the number of seeds dispersed and at all three scales for the proportion of seeds dispersed (Table 3; Fig 4). No other intrinsic or extrinsic drivers had a significant effect on the number or proportion of seeds dispersed (Table 3).

**Table 3:** Results testing for the significance of intrinsic and extrinsic drivers on the quantity component (number and proportion) of seed dispersal effectiveness. Model comparisons evaluated with type II likelihood ratio χ^2^ tests are identified by letter codes after each driver for the number and proportion of seeds, respectively. *P* values in bold indicate a significant effect (α = 0.05).

Driver type	Driver	Number of seeds				Proportion of seeds			
		Pseudo R^2^	χ^2^	df	*P*	Pseudo R^2^	χ^2^	df	*P*
Intrinsic	Individual crop size ^a^^,^^a^	0.17	97.7	1	**< 0.001**		0.86	1	0.35
	Height (scaled) ^b^^,^^b^	0.05	32.2	2	**< 0.001**		5.67	2	0.06
Extrinsic	Vegetation community ^a^^,^^a^		2.9	1	0.40		3.96	1	0.27
	Understory cover ^b^^,^^b^		1.54	2	0.46		0.18	2	0.67
	Time since last fire ^a^^,^^b^	0.05	28.8	1	**< 0.001**	0.11	13.96	2	**< 0.001**
	5 m fruiting neighborhood ^a^^,^^a^	0.02	5.91	1	**0.02**	0.06	6.26	1	**< 0.01**
	10 m fruiting neighborhood ^a^^,^^a^	0.01	4.52	1	**0.03**	0.11	12.5	1	**< 0.001**
	30 m fruiting neighborhood ^a^^,^^a^		0.75	1	0.38	0.08	8.86	1	**< 0.01**

^a^Reduced (driver and random intercept) versus null (intercept only)

^b^Full (driver with a quadratic term and random intercept) versus reduced

Frugivores documented with camera traps at fruiting *L*. *subcoriacea* included the American Robin (*Turdus migratorius* L.), Grey Catbird (*Dumetella carolinensis* L.), Red-headed Woodpecker (*Melanerpes erythrocephalus* L.), and White-eyed Vireo (*Vireo griseus* Boddaert). Given that these four species all have bill gape widths larger than the fruits/seeds of *L*. *subcoriacea*, they are interpreted to be dispersers. The mean occupancies estimated for the American Robin, Red-headed Woodpecker, and White-eyed Vireo within *L*. *subcoriacea* populations across Fort Bragg were 0.35 ± 0.14, 0.23 ± 0.10, and 0.13 ± 0.05, respectively. Bootstrapped confidence intervals for the differences in occupancy estimates among species pairs indicated that frugivore occupancies differed, and were lower than Northern Cardinal occupancy (S3 Table). We could not include Grey Catbird in this comparison because insufficient observations (< 10) during avian surveys prevented us from generating an occupancy map for this species.

### Post-dispersal seed predation

For the seed depot data, there was a difference between the closed and open depots ($\chi_{1\;df}^{2}$ = 116, *P* < 0.001), with open depots having a greater mean proportion of seeds removed (0.65 ± 0.42 vs 0.18 ± 0.28). We removed the closed depots from all further analyses, given the apparent dominant role of rodent granivores in post-dispersal predation. There was a significant difference between a model that included fine woody debris and the null model ($\chi_{1\;df}^{2}$ = 3.91, *P* = 0.048); with higher proportions of seeds predated where the percentage in the substrate was high. No other extrinsic drivers had a significant effect on the proportion of seeds predated collectively by rodents and invertebrates post-dispersal (Table 2). Data and additional details about regression results can be found in S2 and S3 Files.

## Discussion

Information about SDE for species of conservation concern is rarely available to inform management strategies and actions. For *L*. *subcoriacea*, a rare, dioecious shrub of the southeastern USA with low fecundity, we examined the influence of multiple intrinsic and extrinsic drivers on three interconnected processes (dispersal, and pre- and post-dispersal seed predation) that affect the quantitative and qualitative (sub)components of SDE. As reported by others [12], we observed substantial variation in these processes among individuals. We also found that both intrinsic and extrinsic drivers influenced the three processes in diverse ways (S4 Fig). The avian dispersers and one avian seed predator documented at fruiting *L*. *subcoriacea* all significantly differed from one another in their mean occupancy probabilities within *L*. *subcoriacea* populations (range = 0.13–0.42), with the highest mean occupancy estimated for the seed predator. The aggregate consumption of *L*. *subcoriacea* fruits by these disperser species and potentially others not documented by our camera traps is manifest as a higher proportion of dispersed seeds than predated pre-dispersal. While rodent granivores were anticipated to be important post-dispersal seed predators [e.g., 76, 77], we documented that they are also an underappreciated source of pre-dispersal seed predation.

### Pre-dispersal predation

Studies of pre-dispersal seed predation are relatively uncommon compared to studies of post-dispersal predation, and typically focus on insect rather than vertebrate seed predators [e.g., 78]. However, Bell and Clark [79] collected seeds in seed traps in North Carolina over a ~15-year period, examined them for vertebrate seed predation, and documented 18% and 51% pre-dispersal predation of *Cornus florida* L. and *Nyssa sylvatica* Marshall seeds, respectively. Thus, the 28% of *L*. *subcoriacea* seeds that are predated by vertebrates prior to dispersal is within the range documented for these two other drupe-bearing, large-seeded species in the southeastern USA.

Both avian and mammalian seed predators may be attracted to the seeds of *Lindera* spp., which are known to have high lipid and crude protein content [33, 80–82]. However, our methods did not allow us to unequivocally distinguish the magnitude of predation attributable to these two vertebrate guilds. We observed the Northern Cardinal and small arboreal rodents (*Peromyscus* spp.), both well-documented seed predators, at fruiting *L*. *subcoriacea*. Johnson et al. [83] reported that Northern Cardinals commonly crushed the fruits and seeds of *L*. *benzoin* and Smith et al. [56] similarly reported the species as a pre-dispersal seed predator of *L*. *melissifolia*. Given our observations and the high estimated mean occupancy of the Northern Cardinal in *L*. *subcoriacea* populations (0.42 ± 0.11) during August, it is likely that the species is also a *L*. *subcoriacea* seed predator.

Although pre-dispersal seed predation by small arboreal rodents has not been reported for other *Lindera* spp. in North America or Asia, our findings for *L*. *subcoriacea* are not surprising, but rather a heretofore underappreciated source of seed loss. Granivorous rodents are a common component of small mammal communities in many systems, including longleaf pine ecosystems [84]. In our study system *Peromyscus* spp. are the most abundant species of rodent granivore, representing 77–94% of captured individuals and reaching densities of ~50 individuals per 60 x 60 m plot in lowland hardwood habitats [65]. *Peromyscus* spp. exhibit strong preferences for nuts and the seeds of fleshy fruits cleaned of their pulp, such as those deposited post-dispersal, over intact fruits containing seeds [e.g., 85]. However, a positive relationship between *Peromyscus* spp. arboreal activity and density of fleshy fruit producing trees has also been documented in areas without nut producing species [86], which would be characteristic of the wetland habitats occupied by *L*. *subcoriacea*.

The abundance and behavior of both avian and mammalian seed predators are known to vary in response to diverse environmental factors that influence food availability, foraging efficiency, refuge availability, and real and perceived risks of predation [e.g., 87–89]. In the four vegetation communities occupied by *L*. *subcoriacea*, light availability and fire frequency are expected to differ [32, 47, 90] and to influence food- and cover-related resources utilized by seed predators [64, 91]. Although we did not identify differences in pre-dispersal seed predation among vegetation communities or in relation to fire, we did document a negative quadratic relationship with percentage understory cover. Seed predation is generally expected to be greater in areas having high vegetation cover [e.g., 38, 92, 93], but for *L*. *subcoriacea* the highest proportions of pre-dispersal seed predation occurred at intermediate levels of understory cover. Structural complexity can reduce foraging efficiency and is a potential explanation for the observed reduction in pre-dispersal seed predation at high understory cover [e.g., 88], whereas predation risk may limit the activity of seed predators under low understory cover [87].

### Dispersal

If not predated pre-dispersal, the likelihood that *L*. *subcoriacea* seeds are dispersed appears to be comparable to dispersal estimates for *L*. *benzoin*. We estimated that a mean of 69% of seeds are dispersed away from maternal plants and only 3% of seeds fail to disperse. For *L*. *benzoin*, reported dispersal estimates range from 60–90% [33, 34, 94], however neither pre-dispersal predation nor dispersal failure were accounted for in these estimates. Despite rapid and high percentages of removal of fruits by presumed dispersers, *L*. *benzoin*, *C*. *florida*, and *N*. *sylvatica* are also known to suffer dispersal limitation and have small seed shadows [95–97]. Small seed shadows, in addition to source limitation, may also be important for *L*. *subcoriacea*, constraining the number of occupied sites and affecting inter-population dynamics [94].

Both plant height and crop size have been found to be intrinsic drivers of seed dispersal [14, 98]. The relationship between dispersal and plant height is not as well-studied as is that for crop size, and where investigated has exhibited variable results [12]. For *L*. *subcoriacea*, we documented a negative quadratic relationship between plant height and number of seeds dispersed. Previous work has documented a negative quadratic relationship between height and fruit crop size for the species [37], but no relationship between height and crop size was identified for the individuals included in this study (S2 File). Therefore, height had an effect on seed dispersal independent of any effect of individual crop size. For crop size, a recent meta-analysis found a consistent positive relationship with the number, but not the proportion of seeds dispersed [99]. Our findings align with those of the meta-analysis.

Conspecific and heterospecific fruiting neighborhoods can either negatively influence SDE quantity by generating competition for dispersers [e.g., 21–23], or positively influence dispersal quantity through facilitation [e.g., 24,25]. We documented a positive relationship between the number and proportion of seeds dispersed and *L*. *subcoriacea* fruiting neighborhood at nearly all scales examined, suggesting active selection by dispersers. A similar positive conspecific effect has been reported for *L*. *benzoin*, with avian frugivores foraging more frequently on clumped than isolated fruit displays [100]. Although conspecific fruiting neighborhood enhances *L*. *subcoriacea* SDE quantity, it may also potentially reduce SDE quality via reduced dispersal distances [e.g., 26]. Although we did not quantify the number or identity of other fruit-bearing species within *L*. *subcoriacea* fruiting neighborhoods, we do not think that the presence of these species had a large negative effect on *L*. *subcoriacea* seed dispersal, as we documented positive linear relationships between dispersal and both individual crop size and conspecific fruiting neighborhoods. Rather, as suggested by the diet complementation hypothesis [101, 102], it is possible that heterospecific fruit-bearing species within neighborhoods facilitated *L*. *subcoriacea* seed dispersal. This hypothesis proposes that negative frequency-dependent fruit selection for complementary nutrients [e.g., 103, 104] can increase the SDE of rare plants having fruits of relatively higher value compared to common species because their fruits will be consumed at proportionally higher rates [105]. The fruits of *Lindera* spp. are known to be a preferred, high quality, food resource for avian frugivores [33, 106].

As is the case for seed predators, the abundance and behavior of avian seed dispersers are also known to vary in response to diverse environmental factors [39, 107, 108]. Although we did not find any effect of vegetation community or understory cover on dispersal, Moore and Willson [34] documented differences in fruit removal rates between forest interior and gap habitats for *L*. *benzoin*, however the patterns reversed over the fruiting season. We did document a positive linear relationship between time since last fire (TSLF) and the number of seeds dispersed, and a positive quadratic relationship between TSLF and the proportion of seeds dispersed. Fire reduces cover and food resources for avian frugivores by top-killing the woody stems of fleshy-fruited shrubs and setting back recovery to pre-burn size and fruit production for at least 3–4 years in our study system [37, 49, 91].

All of the avian frugivores documented at fruiting *L*. *subcoriacea* individuals by the camera traps are known to consume *L*. *benzoin* and/or *L*. *melissifolia* fruits [34, 100, 109, 110], and are also likely to disperse *L*. *subcoriacea* seeds. These four species are known to swallow whole fruits and regurgitate large seeds [e.g., 83]. Bill gape widths are a limiting factor for consumption of fruits with this handling approach [13]. The bill gape widths of all four of these putative dispersers and the seed predator (i.e. Northern Cardinal) are > 10 mm and exceed the size of *L*. *subcoriacea* fruits [83, 111]. Although not documented by our camera traps, it seems likely that many seasonally frugivorous avian species occurring at our study site during August and having adequately large bill gape widths also consume *L*. *subcoriacea* fruits and disperse seeds.

Although observations of avian frugivore visitation to fruiting *L*. *subcoriacea* are interesting, knowledge of disperser identity provides no explicit information about disperser effectiveness. Nonetheless, some inferences about the likely relative dispersal effectiveness of the four species can be made. For SDE quantity, the number of fruits consumed is expected to increase with avian body mass due to positive relationships between body mass and basal metabolic rate and gut capacity [112]. Therefore, not accounting for any interspecific dietary differences, ordered low to high, White-eyed Vireo, Gray Catbird, Red-headed Woodpecker and American Robin individuals are expected to consume and disperse increasing numbers of fruits/seeds [113]. For SDE quality, we suspect that the Red-headed Woodpecker may be the least effective of the four species, as it commonly inhabits upland savanna communities that would be unsuitable for *L*. *subcoriacea* recruitment [114]. In contrast, the Gray Catbird and White-eyed Vireo primarily move within dense vegetation [114]. In our study system, densely vegetated areas are typically wetland communities that are potentially suitable sites for *L*. *subcoriacea* recruitment [37]. Although, the American Robin is commonly found in upland savannas during the breeding season, their post-breeding shift to a more frugivorous diet is expected to cause greater use of wetland habitats, where fleshy-fruited plant species are abundant, fruit is more likely to be available, and *L*. *subcoriacea* germination and recruitment is possible [47, 91, 115]. Wall et al. [60] estimated post-breeding occupancy maps for these avian species across our study site that align with these natural history-based interpretations of their differential habitat use.

### Post-dispersal predation

When the levels of post-dispersal predation (65%) that we documented for surrogate *L*. *benzoin* seeds are combined with other *L*. *subcoriacea* seed losses, seedling recruitment and population growth rates are potentially limited. The proportion of *L*. *benzoin* seeds that are predated post-dispersal in our study system appears to be comparable to available estimates for the endangered *L*. *melissifolia*, which also has seeds of similar size and presumably composition [116]. Martins et al. [109] used video cameras to document vertebrate visitors at *L*. *melissifolia* seed plots. They documented Northern Cardinals, *Peromyscus* spp., and gray squirrels (*Sciurus carolinensis* Gmelin) consuming seeds within plots. Seed removal from their plots varied among sites and years, with the proportions and rates of removal ranging from 1.0 within 7 days to 0.0 over 68 days. They also recorded higher percentages and rates of seed removal in plots with high (50–100%), than low (0–25%) understory cover. For example, they estimated that the percentage of seeds removed ranged from approximately 55–70% after 15 days in high understory cover conditions. Despite differences in seed presentation, these are comparable proportions of removal over the same duration and in similar vegetation communities and understory cover as our seed removal study. Consequently, it is unlikely that our sous vide heat treatment of *L*. *benzoin* seeds had any substantial effect on our findings due to potential changes in olfactory cues used by rodent granivores.

We did not find any influence of vegetation community type, understory cover, or TSLF on post-dispersal seed predation over two years. For substrate data collected during 2019, we only found a significant positive effect of fine woody debris on seed predation. Overall, these findings were surprising given the abundant evidence that these extrinsic drivers and their interactions can influence rodent granivory [64, 117, 118] and abundance [65] in our study system and more broadly in longleaf pine ecosystems. We estimated mean TSLF and understory cover within a 30 m radius of depot locations, which may have been too coarse of a resolution to identify relationships for small rodent granivores despite successful application elsewhere [e.g., 119]. With regards to fine woody debris, we are unable to speculate why there might be a positive relationship with seed predation except that perhaps fine woody debris was associated with nearby coarse woody debris or standing snags not recorded in our 1 m^2^ microplots. Both coarse woody debris and snags have been shown to be important habitat features for small rodent granivores in the southeastern USA and to influence foraging behavior [41].

Although direct evidence of *in situ* seed predation was commonly found in the form of empty endocarps within depots, we also observed cases where seeds were removed with no evidence of consumption. In our analyses, we assumed all seeds removed from the seed depots were consumed, as opposed to being taken to another location and cached (i.e. secondary dispersal, diplochory). This assumption can lead to overestimation of post-dispersal predation. However, secondary dispersal is difficult to quantify and can be affected by many factors including those related to seeds (e.g., size, nutrient composition, secondary metabolites, strength of protective layers, etc.), granivores (e.g., species, sex, individual behavior, physiological state, etc.), and environmental conditions (e.g., season, lunar cycle, predation risk, etc.) [120].

### Conservation implications and additional research needs

Our estimates for pre- and post-dispersal predation of *L*. *subcoriacea* seeds suggest these two qualitative subcomponents of SDE potentially limit recruitment and population growth rates. This is especially true given the low fecundity, recruitment, and population growth rate documented for the species [37]. They also suggest that active conservation actions may be needed to improve the species conservation status. For example, either *in situ* seeding into predator exclosures or *ex situ* propagation and seedling outplanting may be warranted conservation strategies, if demonstrated to have adequate success [121]. In addition to directly promoting the general goals of population resiliency and redundancy [122], these actions could also be implemented to increase *L*. *subcoriacea* fruiting neighborhoods at scales that enhance SDE quantity. If either approach is employed, care should be taken to use best conservation practices (e.g., harvesting only a subset of available seeds, carefully evaluating the potential suitability of outplanting sites, monitoring the success of conservation actions, etc.) [e.g., 123, 124].

Although our findings suggest *L*. *subcoriacea* seeds are being dispersed by at least several avian species, we do not know with certainty the absolute or relative dispersal effectiveness of the different species in terms of quantity and quality. Given the challenges of observing and tracking birds within the vegetation communities occupied by *L*. *subcoriacea*, it might be useful to instead explore SDE by modeling the fruit consumption, gut retention times, movements, and habitat occupancy of the various species to identify the relative numbers of seeds potentially dispersed and the suitability of dispersal locations for *L*. *subcoriacea* recruitment [e.g., 125–127]. The dispersal information generated by this sort of modeling effort would also be useful for informing models of *L*. *subcoriacea* regional population dynamics that could guide conservation actions such as small population augmentation and population introduction to enhance connectivity [128, 129]. For example, Cipollini et al. [94] examined the importance of long-distance dispersal for patch-specific demography and mean population growth rate of *L*. *benzoin*. Like *L*. *benzoin*, *L*. *subcoriacea* populations are also likely dynamic, with inter-population processes (e.g., immigration and emigration) affecting the viability of populations within sites that vary in suitability over time and space.

Additional studies of *L*. *subcoriacea* SDE quality are also needed. For example, nothing is known about the factors that potentially limit seedling establishment such as light availability, drought, flooding, pathogens, or herbivory, which have been either documented or speculated to influence establishment of *L*. *benzoin* and/or *L*. *melissifolia* [36, 96, 130, 131]. However, low fecundity and high interannual variation in seed production will likely hamper these studies by limiting access to the numbers of seeds needed for robust study designs [37].

## Supporting information

S1 Fig Proportion of all fruits remaining (green) and remaining fruits that are ripe (orange) by date for *Lindera subcoriacea* individuals (*n* = 26) monitored 2017–2019 on Fort Bragg, NC, USA. Boxes denote the interquartile range, horizontal solid lines in boxes denote the median, vertical bars represent ± 1.5 times the interquartile range, and dots are outliers. Ticks on horizonatal axis represent the date: month (Jul = July, Aug = August) and date (beginning of a 7 day period over which data are summarized).
(TIFF)

S2 Fig Histogram of the proportion of seeds predated prior to dispersal for *Lindera subcoriacea* individuals (*n* = 26) surveyed 2017–2019. (TIFF)

S3 Fig Histogram of the proportion of seeds dispersed (conditional on not being predated) for *Lindera subcoriacea* individuals (*n* = 26) surveyed 2017–2019. (TIFF)

S4 Fig Graphical abstract showing effects of intrinsic and extrinsic drivers on *Lindera subcoriacea* SDE quantity and quality. Cells shaded gray identify examined relationships between processes (second column rows) and drivers (remaining columns to the right). Straight arrows identify linear relationships and curvilinear arrows identify quadratic relationships (positive and negative) between the processes and drivers for both the numbers (#) and percentages (%) of seeds. Where no arrows are displayed, no significant relationships between the process and drivers were identified.
(TIF)

S1 Table Correlation matrix for the intrinsic and extrinsic drivers on *Lindera subcoriacea* pre-dispersal seed predation and dispersal. (DOCX)

S2 Table Correlation matrix for the extrinsic drivers on *Lindera benzoin* post-dispersal seed predation. (DOCX)

S3 Table Differences in mean occupancy estimates within *Lindera subcoriacea* populations between avian species pairs and bootstrapped 95% confidence intervals. Confidence intervals that do not overlap with zero are significantly different.
(DOCX)

S1 File Seed trap data. (CSV)

S2 File Regression results. (DOCX)

S3 File Seed depot data. (CSV)

## References

[1] Nathan R, Muller-Landau HC. Spatial patterns of seed dispersal, their determinants and consequences for recruitment. Trends Ecol Evol. 2000;15: 278–285. doi: 10.1016/s0169-5347(00)01874-7 10856948

[2] Levin SA, Muller-Landau HC, Nathan R, Chave J. The ecology and evolution of seed dispersal: a theoretical perspective. Annu Rev Ecol Evol Syst. 2003;34: 575–604. doi: 10.1146/annurev.ecolsys.34.011802.132428

[3] Schupp EW, Jordano P, Gómez JM. Seed dispersal effectiveness revisited: A conceptual review. New Phytol. 2010;188: 333–353. doi: 10.1111/j.1469-8137.2010.03402.x 20673283

[4] Schupp EW. Quantity, quality and the effectiveness of seed dispersal by animals. In Frugivory and seed dispersal: ecological and evolutionary aspects. Vegetatio. 1993;107/108: 15–29. Available: https://link-springer-com.ezproxy.library.wur.nl/content/pdf/10.1007%2FBF00052209.pdf

[5] Blendinger PG. Functional equivalence in seed dispersal effectiveness of *Podocarpus parlatorei* in Andean fruit-eating bird assemblages. Front Ecol Evol. 2017;5. doi: 10.3389/fevo.2017.00057

[6] Jordano P, Schupp EW. Seed disperser effectiveness: the quantity component and patterns of seed rain for *Prunus mahaleb*. Ecol Monogr. 2000;70: 591. doi: 10.2307/2657187

[7] Traveset A, Verdú M. A meta-analysis of the effect of gut treatment on seed germination. Seed dispersal and frugivory: Ecology, evolution, and conservation. Wallingford, UK: CAB International; 2002. pp. 339–350.

[8] Traveset A, Robertson A, Rodriguez-Pérez J. A review on the role of endozoochory in seed germination. In: Dennis A, Schupp E, Green R, Westcott D, editors. Seed dispersal: theory and its application in a changing world. Oxforshire, UK: CAB International; 2007. pp. 78–103. doi: 10.1079/9781845931650.0078

[9] Soltani E, Baskin CC, Baskin JM, Heshmati S, Mirfazeli MS. A meta-analysis of the effects of frugivory (endozoochory) on seed germination: role of seed size and kind of dormancy. Plant Ecol. 2018;219: 1283–1294. doi: 10.1007/s11258-018-0878-3

[10] Clark JS, Beckage B, Camill P, Cleveland B, HilleRisLambers J, Lichter J, et al. Interpreting recruitment limitation in forests. Am J Bot. 1999;86: 1–16. doi: 10.2307/2656950 21680341

[11] Côrtes MC, Uriarte M. Integrating frugivory and animal movement: A review of the evidence and implications for scaling seed dispersal. Biol Rev. 2013;88: 255–272. doi: 10.1111/j.1469-185X.2012.00250.x 23136896

[12] Schupp EW, Zwolak R, Jones LR, Snell RS, Beckman NG, Aslan C, et al. Intrinsic and extrinsic drivers of intraspecific variation in seed dispersal are diverse and pervasive. AoB Plants. 2019;11: 1–20. doi: 10.1093/aobpla/plz067 31857875 PMC6914678

[13] Wheelwright N. Fruit-size, gape width, and the diets of fruit-eating birds. Ecology. 1985;66: 808–818.

[14] Flörchinger M, Braun J, Böhning-Gaese K, Schaefer HM. Fruit size, crop mass, and plant height explain differential fruit choice of primates and birds. Oecologia. 2010;164: 151–161. doi: 10.1007/s00442-010-1655-8 20490552

[15] Carlo TA, Collazo JA, Groom MJ. Avian fruit preferences across a Puerto Rican forested landscape: Pattern consistency and implications for seed removal. Oecologia. 2003;134: 119–131. doi: 10.1007/s00442-002-1087-1 12647189

[16] Figueroa-Esquivel E, Puebla-Olivares F, Godínez-Álvarez H, Núñez-Farfán J. Seed dispersal effectiveness by understory birds on *Dendropanax arboreus* in a fragmented landscape. Biodivers Conserv. 2009;18: 3357–3365. doi: 10.1007/s10531-009-9645-z

[17] Jones LR, Duke-Sylvester SM, Leberg PL, Johnson DM. Closing the gaps for animal seed dispersal: Separating the effects of habitat loss on dispersal distances and seed aggregation. Ecol Evol. 2017;7: 5410–5425. doi: 10.1002/ece3.3113 28770078 PMC5528214

[18] Saavedra F, Hensen I, Beck SG, Böhning-Gaese K, Lippok D, Töpfer T, et al. Functional importance of avian seed dispersers changes in response to human-induced forest edges in tropical seed-dispersal networks. Oecologia. 2014;176: 837–848. doi: 10.1007/s00442-014-3056-x 25182931

[19] Bovo AAA, Ferraz KMPMB, Magioli M, Alexandrino ER, Hasui É, Ribeiro MC, et al. Habitat fragmentation narrows the distribution of avian functional traits associated with seed dispersal in tropical forest. Perspect Ecol Conserv. 2018;16: 90–96. doi: 10.1016/j.pecon.2018.03.004

[20] Snell RS, Beckman NG, Fricke E, Loiselle BA, Carvalho CS, Jones LR, et al. Consequences of intraspecific variation in seed dispersal for plant demography, communities, evolution and global change. AoB Plants. 2019;11: 1–19. doi: 10.1093/aobpla/plz016 31346404 PMC6644487

[21] Denslow JS. Fruit removal rates from aggregated and isolated bushes of the red elderberry, *Sambucus pubens*. Can J Bot. 1987;65: 1229–1235. doi: 10.1139/b87-170

[22] Rumeu B, Álvarez-Villanueva M, Arroyo JM, González-Varo JP. Interspecific competition for frugivores: population-level seed dispersal in contrasting fruiting communities. Oecologia. 2019;190: 605–617. doi: 10.1007/s00442-019-04434-9 31197480

[23] Smith AD, McWilliams SR. Fruit removal rate depends on neighborhood fruit density, frugivore abundance, and spatial context. Oecologia. 2014;174: 931–942. doi: 10.1007/s00442-013-2834-1 24305861

[24] García D, Zamora R, Gómez JM, Hódar JA. Frugivory at Juniperus communis depends more on population characteristics than on individual attributes. J Ecol. 2001;89: 639–647.

[25] Albrecht J, Bohle V, Berens DG, Jaroszewicz B, Selva N, Farwig N. Variation in neighbourhood context shapes frugivore-mediated facilitation and competition among co-dispersed plant species. J Ecol. 2015;103: 526–536. doi: 10.1111/1365-2745.12375

[26] Carlo TA, Morales JM. Inequalities in fruit-removal and seed dispersal: Consequences of bird behaviour, neighbourhood density and landscape aggregation. J Ecol. 2008;96: 609–618. doi: 10.1111/j.1365-2745.2008.01379.x

[27] Jansen PA, Visser MD, Joseph Wright S, Rutten G, Muller-Landau HC. Negative density dependence of seed dispersal and seedling recruitment in a Neotropical palm. Ecol Lett. 2014;17: 1111–1120. doi: 10.1111/ele.12317 25039608

[28] Clark JS, Dietze M, Chakraborty S, Agarwal PK, Ibanez I, LaDeau S, et al. Resolving the biodiversity paradox. Ecol Lett. 2007;10: 647–659. doi: 10.1111/j.1461-0248.2007.01041.x 17594418

[29] Comita LS, Muller-Landau HC, Aguilar S, Hubbell SP. Asymmetric density dependence shapes species abundances in a tropical tree community. Science (80-). 2010;329: 330–332. doi: 10.1126/science.1190772 20576853

[30] Dalrymaple S, Banks E, Stewart G, Pullin A. A meta-analysis of threatened plant reintroductions from across the globe. Plant reintroduction in a changing climate. Washington, DC: Island Press; 2012. pp. 31–50.

[31] Rabinowitz D. The seven forms of rarity. In: Synge H, editor. The biological aspects of rare plant conservation. New York: Wiley; 1981.

[32] Wall WA, Hohmann MG, Walker AS, Gray JB. Sex ratios and population persistence in the rare shrub *Lindera subcoriacea* Wofford. Plant Ecol. 2013;214: 1105–1114. doi: 10.1007/s11258-013-0234-6

[33] Stiles EW. Patterns of fruit presentation and seed dispersal in bird-disseminated woody plants in the eastern deciduous forest. Am Nat. 1980;116: 670–688. doi: 10.1086/283657

[34] Moore LA, Willson MF. The effect of microhabitat, spatial distribution, and display size on dispersal of *Lindera benzoin* by avian frugivores. Can J Bot. 1982;60: 557–560. doi: 10.1139/b82-076

[35] Hawkins TS, Walck JL, Hidayati SN. Seed ecology of *Lindera melissifolia* (Lauraceae) as it relates to rarity of the species. J Torrey Bot Soc. 2011;138: 298–307. doi: 10.3159/TORREY-D-11-00021.1

[36] Cipollini ML, Culberson J, Whigham D, Johnson K, Knight T, O’Neill J. Spatial and temporal patterns of sexual dimorphism and sex ratio in *Lindera benzoin* L. (Lauraceae)1,2. J Torrey Bot Soc. 2013;140: 280–299. doi: 10.3159/TORREY-D-13-00013.1

[37] Wall WA, Walker AS, Gray JB, Hohmann MG. Fire effects on the vital rates and stochastic population growth rate of the rare shrub *Lindera subcoriacea* Wofford. Plant Ecol. 2021;222: 119–131. doi: 10.1007/s11258-020-01092-3

[38] Anderson CS, Cady AB, Meikle DB. Effects of vegetation structure and edge habitat on the density and distribution of white-footed mice (*Peromyscus leucopus*) in small and large forest patches. Can J Zool. 2003;81: 897–904. doi: 10.1139/z03-074

[39] McCabe JD, Olsen BJ. Tradeoffs between predation risk and fruit resources shape habitat use of landbirds during autumn migration. Auk. 2015;132: 903–913. doi: 10.1642/AUK-14-213.1

[40] Valentine LE, Schwarzkopf L, Johnson CN. Effects of a short fire-return interval on resources and assemblage structure of birds in a tropical savanna. Austral Ecol. 2012;37: 23–34. doi: 10.1111/j.1442-9993.2011.02244.x

[41] Loeb SC. Responses of small mammals to coarse woody debris in a Southeastern pine forest. J Mammal. 1999;80: 460–471. doi: 10.2307/1383293

[42] Alcaintara JM, Rey PJ, Valera F, Sainchez-lafuente AM, Gutierrez JE. Habitat alteration and plant intra-specific competition for seed dispersers. An Example with *Olea europaea* var. *sylvestris*. Oikos. 1997;79: 291–300.

[43] Izhaki Ido. The role of fruit traits in determining fruit removal in east Mediterranean ecosystems. In: Levey DJ, Silva WR, Galetti M, editors. Seed dispersal and frugivory: ecology, evolution and conservation Third International Symposium-Workshop on Frugivores and Seed Dispersal, São Pedro, Brazil, 6–11 August 2000. Wallingford, UK: CAB International; 2001. pp. 161–174. doi: 10.1079/9780851995250.0161

[44] Griffith G, Omernik J, Comstock J, Schafale M, NcNab W, Lenat D, et al. Ecoregions of North Carolina and South Carolina (U.S. Geological Survey Map). Reston, VA; 2002.

[45] Wells B, Shunk I. The vegetation and habitat factors of the coarser sands of the North Carolina Coastal Plain: an ecological study. Ecol Monogr. 1931;1: 465–520. doi: 10.2307/1943080

[46] Stambaugh MC, Guyette RP, Marschall JM. Longleaf pine (*Pinus palustris* Mill.) fire scars reveal new details of a frequent fire regime. J Veg Sci. 2011;22: 1094–1104. doi: 10.1111/j.1654-1103.2011.01322.x

[47] Sorrie BA, Gray JB, Crutchfield PJ. The vascular flora of the longleaf pine ecosystem of Fort Bragg and Weymouth Woods, North Carolina. Castanea. 2006;71: 129–161. doi: 10.2179/05-02.1

[48] Just MG, Hohmann MG, Hoffmann WA. Where fire stops: vegetation structure and microclimate influence fire spread along an ecotonal gradient. Plant Ecol. 2016;217: 631–644. doi: 10.1007/s11258-015-0545-x

[49] Grady JM, Hoffmann WA. Caught in a fire trap: recurring fire creates stable size equilibria in woody resprouters. Ecology. 2012;93: 2052–60. Available: http://www.ncbi.nlm.nih.gov/pubmed/23094377 doi: 10.1890/12-0354.1 23094377

[50] Schafer JL, Just MG. Size dependency of post-disturbance recovery of multi-stemmed resprouting trees. PLoS One. 2014;9: e105600. doi: 10.1371/journal.pone.0105600 25144236 PMC4140811

[51] Wall WA, Hohmann MG, Just MG, Hoffmann WA. Characterizing past fire occurrence in longleaf pine ecosystems with the Mid-Infrared Burn Index and a Random Forest classifier. For Ecol Manage. 2021;500: 119635. doi: 10.1016/j.foreco.2021.119635

[52] Schafale M. Guide to the natural communities of North Carolina: fourth approximation. Raleigh, NC: Department of Environment, Health, and Natural Resources, North Carolina Natural Heritage Program, Division of Parks and Recreation; 2012. Available: http://cvs.bio.unc.edu/pubs/4thApproximationGuideFinalMarch2012.pdf

[53] Sinnott-Armstrong MA, Downie AE, Federman S, Valido A, Jordano P, Donoghue MJ. Global geographic patterns in the colours and sizes of animal-dispersed fruits. Glob Ecol Biogeogr. 2018;27: 1339–1351. doi: 10.1111/geb.12801

[54] Lei B, Cui J, Newman C, Buesching CD, Xie Z, MacDonald DW, et al. Seed dispersers shape the pulp nutrients of fleshy-fruited plants. Proc R Soc B Biol Sci. 2021;288. doi: 10.1098/rspb.2021.0817 34157866 PMC8220262

[55] Schaefer HM, Valido A, Jordano P. Birds see the true colours of fruits to live off the fat of the land. Proc R Soc B Biol Sci. 2014;281. doi: 10.1098/rspb.2013.2516 24403330 PMC3896014

[56] Smith CGI, Hamel PBPB, Devall MSMS, Schiff NM, III CS. Hermit thrush is the first observed dispersal agent for pondberry (*Lindera melissifolia*). Castanea. 2004;69: 1–8. doi: 10.2179/0008-7475(2004)069&lt;0001:HTITFO&gt;2.0.CO;2

[57] Craves J. Morphological traits of common autumn-ripening bird-dispersed fruits in southeastern Michigan. University of Michigan. 2017.

[58] Davidar P, Morton ES. The relationship between fruit crop sizes and fruit removal rates by birds. Ecology. 1986;67: 262–265. doi: 10.2307/1938529

[59] Niesenbaum RA. Light or pollen—seasonal limitations on female reproductive success in the understory shrub *Lindera Benzoin*. J Ecol. 1993;81: 315. doi: 10.2307/2261501

[60] Hohmann M, Wall W, Wilcox M. Canopy cover and understory heterogeneity effects on post-breeding avian occupancy and species richness in a longleaf pine ecosystem under active fire management [To be submitted to Ornithological Applications]. 2022.

[61] MacKenzie DI, Nichols JD, Lachman GB, Droege S, Royle AA, Langtimm CA. Estimating site occupancy rates when detection probabilities are less than one. Ecology. 2002;83: 2248–2255. doi: 10.1890/0012-9658(2002)083[2248:ESORWD]2.0.CO;2

[62] Fiske IJ, Chandler RB. Unmarked: An R package for fitting hierarchical models of wildlife occurrence and abundance. J Stat Softw. 2011;43: 1–23. doi: 10.18637/jss.v043.i10

[63] Bent A, Austin O. Life histories of North American cardinals, grosbeaks, buntings, towhees, finches, sparrows, and allies. Bull United States Natl Museum. 1968; 237.

[64] Krall JS, Hohmann MG, Fraterrigo JM. Contingent fire effects on granivore removal of exotic woody plant seeds in longleaf pine savannas. Biol Invasions. 2014;16: 1055–1068.

[65] Sasmal I, Deperno CS, Swingen MB, Moorman CE. Influence of vegetation type and prescribed fire on Peromyscus abundance in a longleaf pine ecosystem. Wildl Soc Bull. 2017;41: 49–54. doi: 10.1002/wsb.740

[66] Frost CC, Wilds S. Presettlement vegetation and natural fire regimes of Fort Bragg, North Carolina. [Report prepared for Endangered Species Branch, Natural Resources Division XVIII Airborne Corps, Fort Bragg, North Carolina]. Fort Bragg, NC; 2005.

[67] Craig MT, Orrock JL, Brudvig LA. Edge-mediated patterns of seed removal in experimentally connected and fragmented landscapes. Landsc Ecol. 2011;26: 1373–1381. doi: 10.1007/s10980-011-9650-y

[68] Wofford BE. A new Lindera (Lauraceae) from North America. J Arnold Arboretum. 1983;64: 325–331. doi: 10.5962/bhl.part.27407

[69] Dormann CF, Elith J, Bacher S, Buchmann C, Carl G, Carré G, et al. Collinearity: A review of methods to deal with it and a simulation study evaluating their performance. Ecography (Cop). 2013;36: 27–46. doi: 10.1111/j.1600-0587.2012.07348.x

[70] Bates D, Mächler M, Bolker B, Walker S. Fitting linear mixed-effects models using lme4. J Stat Softw. 2015;67: 1–48. doi: 10.18637/jss.v067.i01

[71] Chambers J, Hastie T. Statistical models in S: Wadsworth & Brooks/Cole computer science series. Boca Raton, FL: CRC Press; 1992.

[72] Bolker BM, Brooks ME, Clark CJ, Geange SW, Poulsen JR, Stevens MHH, et al. Generalized linear mixed models: a practical guide for ecology and evolution. Trends Ecol Evol. 2009;24: 127–135. doi: 10.1016/j.tree.2008.10.008 19185386

[73] Bolker BM. Linear and generalized linear mixed models. In: Fox G, Negrete-Yankelevich S, Sosa V, editors. Ecological statistics: contemporary theory and application. New York, NY: Oxford University Press; 2015. pp. 309–334.

[74] R Core Team. R (4.1.0): A language and environment for statistical computing. R Foundation for Statistical Computing, Vienna, Austria. http://www.R-project.org/. Vienna, Austria: R Foundation for Statistical Computing; 2021. Available: http://www.r-project.org

[75] McFaddenn D. Conditional logit analysis of qualitative choice behavior. In: Zarembka P, editor. Frontiers in econometrics. New York, NY: Academic Press; 1973. pp. 105–142. doi: 10.1080/07373937.2014.997882

[76] Hulme PE, Benkman CW. Granivory. In: Herrera CM, Pelmyr O, editors. Plant animal interactions: An evolutionary approach. Hoboken, NJ, USA: Wiley-Blackwell; 2002. pp. 185–208.

[77] Hulme PE. Seed eaters: seed dispersal, destruction and demography. In: Levey DJ, Silva M, Galetti M, editors. Seed dispersal and frugivory: Ecology, evolution and conservation. Wallingford, UK: CAB International; 2002. pp. 257–273.

[78] Kolb A, Ehrlén J, Eriksson O. Ecological and evolutionary consequences of spatial and temporal variation in pre-dispersal seed predation. Perspect Plant Ecol Evol Syst. 2007;9: 79–100. doi: 10.1016/j.ppees.2007.09.001

[79] Bell DM, Clark JS. Seed predation and climate impacts on reproductive variation in temperate forests of the southeastern USA. Oecologia. 2016;180: 1223–1234. doi: 10.1007/s00442-015-3537-6 26747267

[80] Billingsley B, Arner D. The nutritive value and digestibility of some winter foods of the eastern wild turkey. J Wildl Manage. 1970;34: 176–182.

[81] Bajracharya D. Nutritive values of Nepalese edible wild fruits. Zeitschrift für Leb und Forsch. 1980;171: 363–366. doi: 10.1007/BF01087135 7445758

[82] Conway C, Eddleman W, Simpson K. Seasonal changes in fatty acid composition of the Wood Thrush. Condor. 1994;96: 791–794.

[83] Johnson RA, Willson MF, Thompson JN, Bertin RI. Nutritional values of wild fruits and consumption by migrant frugivorous birds. Ecology. 1985;66: 819–827.

[84] Means D. Vertebrate faunal diversity of longleaf pine ecosystems. In: Jose S, Jokela E, Miller D, editors. The longleaf pine ecosystem: ecology, silviculture, and restoration. New York: Springer; 2006. pp. 157–213.

[85] Perea R, San Miguel A, Gil L. Disentangling factors controlling fruit and seed removal by rodents in temperate forests. Seed Sci Res. 2011;21: 227–234. doi: 10.1017/S0960258511000122

[86] McShea WJ, Gilles AB. A comparison of traps and fluorescent powder to describe foraging for mast by *Peromyscus leucopus*. J Mammal. 1992;73: 218–222. Available: https://www.jstor.org/stable/1381886

[87] Stanback MT, Powell EM. Predator vocalizations affect foraging trade-offs of Northern Cardinals. Wilson J Ornithol. 2010;122: 168–173. doi: 10.1676/09-052.1

[88] Zwolak R, Pearson DE, Ortega YK, Crone EE. Mechanisms driving postfire abundance of a generalist mammal. Can J Zool. 2012;90: 51–60. doi: 10.1139/Z11-111

[89] Jorge MH, Garrison EP, Conner LM, Cherry MJ. Fire and land cover drive predator abundances in a pyric landscape. For Ecol Manage. 2020;461: 117939. doi: 10.1016/j.foreco.2020.117939

[90] Drewa PB, Platt WJ, Moser EB, Rouge B. Fire effects on resprouting of shrubs in headwaters of southeastern longleaf pine savannas. Ecology. 2002;83: 755–767.

[91] Lashley M, Chitwood M, Harper C, DePerno C, Moorman C. Variability in fire prescriptions to promote wildlife foods in the longleaf pine ecosystem. Fire Ecol. 2015;11: 62–79. doi: 10.4996/fireecology.1103062

[92] Bowers MA, Dooley JL. Predation hazard and seed removal by small mammals: microhabitat versus patch scale effects. Oecologia. 1993;94: 247–254. doi: 10.1007/BF00341324 28314039

[93] Jacob SA, Matter SF, Cameron GN. Interactive effects of vegetation and illumination on foraging behavior of white-footed mice (*Peromyscus leucopus*). J Mammal. 2017;98: 804–814. doi: 10.1093/jmammal/gyx012

[94] Cipollini ML, Wallace-Senft DA, Whigham DF. A model of patch dynamics, seed dispersal, and sex ratio in the dioecious shrub Lindera benzoin (Lauraceae). J Ecol. 1994;82: 621. doi: 10.2307/2261269

[95] Matlack GR. Plant species migration in a mixed-history forest landscape in eastern North America. Ecology. 1994;75: 1491–1502. doi: 10.2307/1937472

[96] McEuen AB, Curran LM. Plant recruitment bottlenecks in temperate forest fragments: Seed limitation and insect herbivory. Plant Ecol. 2006;184: 297–309. doi: 10.1007/s11258-005-9074-3

[97] Clark JS, Macklin E, Wood L. Stages and spatial scales of recruitment limitation in southern Appalachian forests. Ecol Monogr. 1998;68: 213–235. doi: 10.1890/0012-9615(1998)068[0213:SASSOR]2.0.CO;2

[98] Hughes L, Dunlop M, French K, Leishman MR, Rice B, Rodgerson L, et al. Predicting dispersal spectra: A minimal set of hypotheses based on plant attributes. J Ecol. 1994;82: 933. doi: 10.2307/2261456

[99] Palacio FX, Ordano M. The strength and drivers of bird-mediated selection on fruit crop size: A meta-analysis. Front Ecol Evol. 2018;6. doi: 10.3389/fevo.2018.00018

[100] Malmborg PK, Willson MF. Foraging ecology of avian frugivores and some consequences for seed dispersal in an Illinois woodlot. Condor. 1988;90: 173–186. doi: 10.2307/1368446

[101] Morán-López T, Carlo TA, Amico G, Morales JM. Diet complementation as a frequency-dependent mechanism conferring advantages to rare plants via dispersal. Funct Ecol. 2018;32: 2310–2320. doi: 10.1111/1365-2435.13152

[102] Morán-López T, Carlo TA, Morales JM. The role of frugivory in plant diversity maintenance–a simulation approach. Ecography (Cop). 2018;41: 24–31. doi: 10.1111/ecog.03220

[103] Murphy ME. Dietary complementation by wild birds: Considerations for field studies. J Biosci. 1994;19: 355–368. doi: 10.1007/BF02703173

[104] Whelan CJ, Schmidt KA, Steele BB, Quinn W, Dilger S. Are bird-consumed fruits complementary resources? Oikos. 1998;83: 195–205. Available: https://www.jstor.org/stable/3546561

[105] Blendinger PG, Martín E, Osinaga Acosta O, Ruggera RA, Aráoz E. Fruit selection by Andean forest birds: Influence of fruit functional traits and their temporal variation. Biotropica. 2016;48: 677–686. doi: 10.1111/btp.12329

[106] White D, Stiles E. Bird dispersal of fruits of species introduced into eastern North America. Candian J Bot. 1992;70: 1689–1696.

[107] Breininger D, Schmalzer P. Effects of fire and disturbance on plants and birds in a Florida oak/palmetto scrub community. Am Midl Nat. 1990;123: 64–74.

[108] Ruhl PJ, Delancey CD, Dunning JB. Roost preference, postfledging habitat use, and breeding phenology of adult female Worm-eating Warblers (*Helmitheros vermivorum*) on the breeding grounds. Wilson J Ornithol. 2018;130: 397–409. doi: 10.1676/16-222.1

[109] Martins AM, Abilio FM, Oliveira PG de, Feltrin RP, Scheffer Alves de Lima F, de O. Antonelli P, et al. Pondberry (*Lindera melissifolia*, Lauraceae) seed and seedling dispersers and predators. Glob Ecol Conserv. 2015;4: 358–368. doi: 10.1016/j.gecco.2015.07.008

[110] Martin AC, Zim HS, Nelson AL. American wildlife & plants: a guide to wildlife food habits: the use of trees, shrubs, weeds, and herbs by birds and mammals of the United States. Reprint ed. Mineola, NY: Dover Publications; 1961.

[111] Crowell KL. Reduced interspecific competition among the birds of Bermuda. Ecology. 1962;43: 75–88. doi: 10.2307/1932042

[112] McNab BK. Ecological factors affect the level and scaling of avian BMR. Comp Biochem Physiol—A Mol Integr Physiol. 2009;152: 22–45. doi: 10.1016/j.cbpa.2008.08.021 18805499

[113] Dunning Jr. J. CRC handbook of avian body masses. 2nd. CRC Press; 2008.

[114] Allen JC, Krieger SM, Walters JR, Collazo JA. Associations of breeding birds with fire-influenced and riparian-upland gradients in a longleaf pine ecosystem. Auk. 2006;123: 1110–1128. doi: 10.1642/0004-8038(2006)123[1110:AOBBWF]2.0.CO;2

[115] Greenberg CH, Levey DJ, Kwit C, McCarty JP, Pearson SF, Sargent S, et al. Long-term patterns of fruit production in five forest types of the South Carolina upper coastal plain. J Wildl Manage. 2012;76: 1036–1046. doi: 10.1002/jwmg.343

[116] Connor K, Schaefer G, Donahoo J, Devall M, Gardiner E, Hawkins T, et al. Development, fatty acid composition, and storage of drupes and seeds from the endangered pondberry (*Lindera melissifolia*). Biol Conserv. 2007;137: 489–496. doi: 10.1016/j.biocon.2007.03.011

[117] Willis JL, Schnake DK, DePerno CS, Lashley MA, Wetzstein B, Yow J. Tree encroachment impacts on seed predator selection and seedling establishment in degraded pine woodlands. Appl Veg Sci. 2021;24: 1–11. doi: 10.1111/avsc.12570

[118] Willis JL, Schnake DK, Wetzstein B, Yow J, Guinto D, Ulrich S, et al. Seed depredation negates the benefits of midstory hardwood removal on longleaf pine seedling establishment. Restor Ecol. 2019;27: 1064–1072. doi: 10.1111/rec.12951

[119] Roberts SL, van Wagtendonk JW, Miles AK, Kelt DA, Lutz JA. Modeling the effects of fire severity and spatial complexity on small mammals in Yosemite National Park, California. Fire Ecol. 2008;4: 83–104. doi: 10.4996/fireecology.0402083

[120] Lichti NI, Steele MA, Swihart RK. Seed fate and decision-making processes in scatter-hoarding rodents. Biol Rev. 2015;92: 474–504. doi: 10.1111/brv.12240 26587693

[121] Kunz M, Buchanan MF, Randall JL, Wall WA, Hohmann MG. Life cycle, vegetative propagation, and reintroduction of federally endangered rough-leaved loosestrife, *Lysimachia asperulifolia*. Castanea. 2014;79: 18–26. doi: 10.2179/13-007

[122] Shaffer M, Stein B. Safeguarding our precious heritage. In: Stein B, Kutner L, Adams J, editors. Precious heritage: the status of biodiversity in the United States. New York, NY: Oxford University Press; 2000. pp. 301–322.

[123] Maschinski J, Albrecht MA. Center for Plant Conservation’s best practice guidelines for the reintroduction of rare plants. Plant Divers. 2017;39: 390–395. doi: 10.1016/j.pld.2017.09.006 30159534 PMC6112315

[124] Ames GM, Wall WA, Hohmann MG, Wright JP. Functional trait similarity predicts survival in rare plant reintroductions. Ecol Appl. 2020;30: 1–9. doi: 10.1002/eap.2087 32017309

[125] Rehm E, Fricke E, Bender J, Savidge J, Rogers H. Animal movement drives variation in seed dispersal distance in a plant-animal network. Proc R Soc B Biol Sci. 2019;286. doi: 10.1098/rspb.2018.2007 30963874 PMC6367185

[126] Morales JM, Morán López T. Mechanistic models of seed dispersal by animals. Oikos. 2021; 1–16. doi: 10.1111/oik.08328

[127] Just MG, Wall WA, Huskins S, Hohmann MG. Effects of landscape heterogeneity and disperser movement on seed dispersal. [in revision].

[128] MacArthur R, Wilso E. The theory of island biogeography. Princeton, NJ: Princeton University Press; 1967.

[129] Hanksi I. Spatially realistic theory of metapopulation ecology. Naturwissenschaften. 2001;88: 372–381. doi: 10.1007/s001140100246 11688412

[130] Aleric KM, Kirkman LK. Growth and photosynthetic responses of the federally endangered shrub, *Lindera melissifolia* (Lauraceae), to varied light environments. Am J Bot. 2005;92: 682–689. doi: 10.3732/ajb.92.4.682 21652446

[131] Hawkins TS, Schiff NM, Leininger TD, Gardiner ES, Devall MS, Hamel PB, et al. Growth and intraspecific competitive abilities of the dioecious *Lindera melissifolia* (Lauraceae) in varied flooding regimes. J Torrey Bot Soc. 2009;136: 91–101. doi: 10.3159/08-RA-049R1.1

