# Supplementary figures and images for: Multiple intrinsic and extrinsic drivers influence the quantity and quality components of seed dispersal effectiveness in the rare shrub Lindera subcoriacea
Source: PLoS One. 2023 Mar 31;18(3):e0283810. doi: 10.1371/journal.pone.0283810 (PMC10065295; doi:10.1371/journal.pone.0283810)

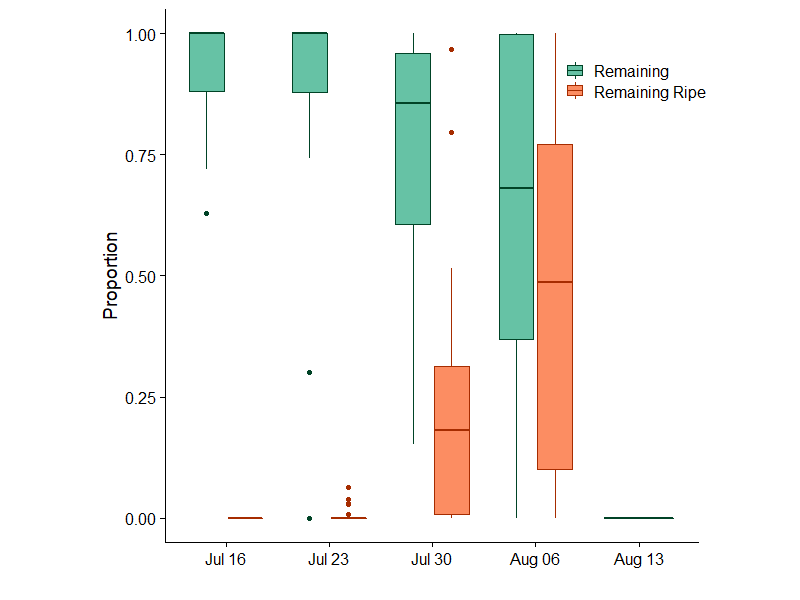

Supplement: S1 Fig — Boxes denote the interquartile range, horizontal solid lines in boxes denote the median, vertical bars represent ± 1.5 times the interquartile range, and dots are outliers. Ticks on horizonatal axis represent the date: month (Jul = July, Aug = August) and date (beginning of a 7 day period over which data are summarized). (TIFF) [file pone.0283810.s001.tiff]

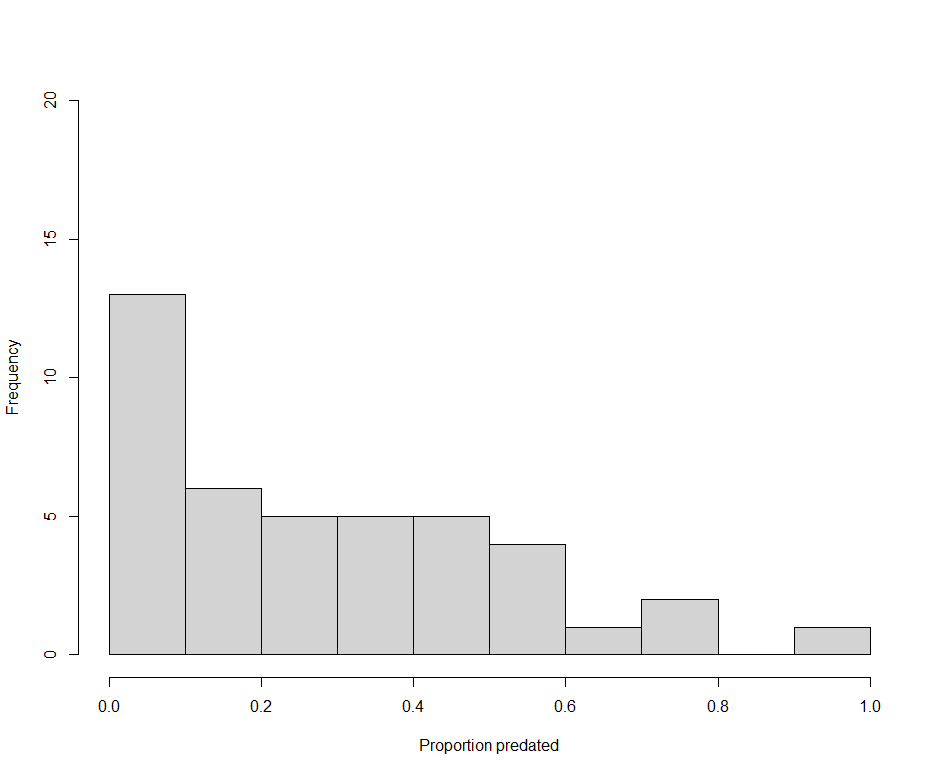

Supplement: S2 Fig — (TIFF) [file pone.0283810.s002.tiff]

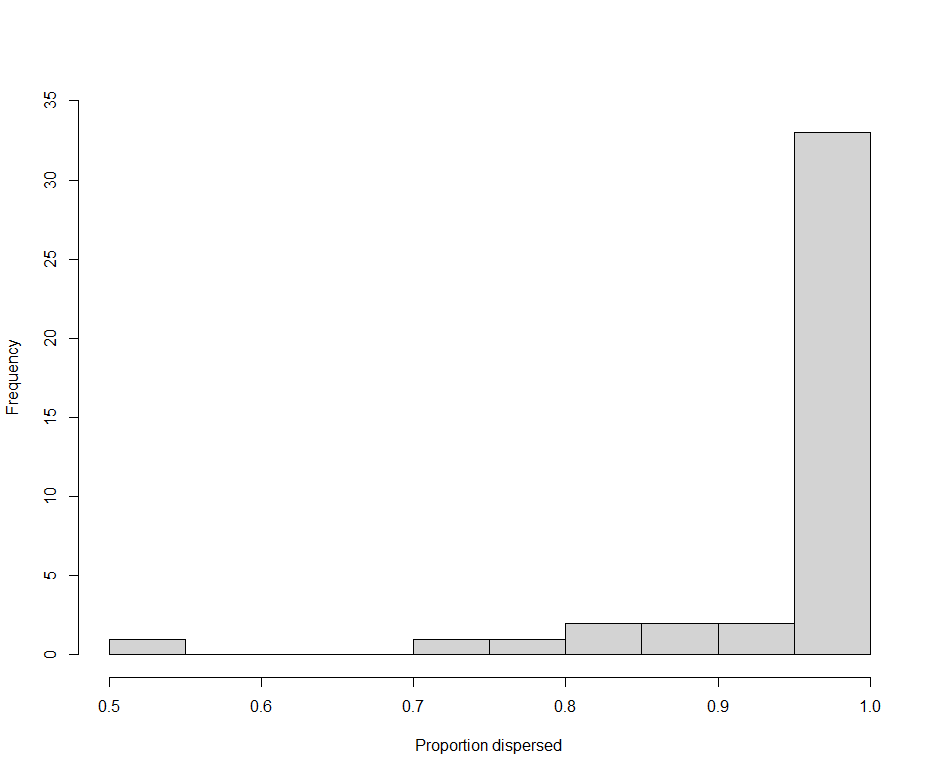

Supplement: S3 Fig — (TIFF) [file pone.0283810.s003.tiff]

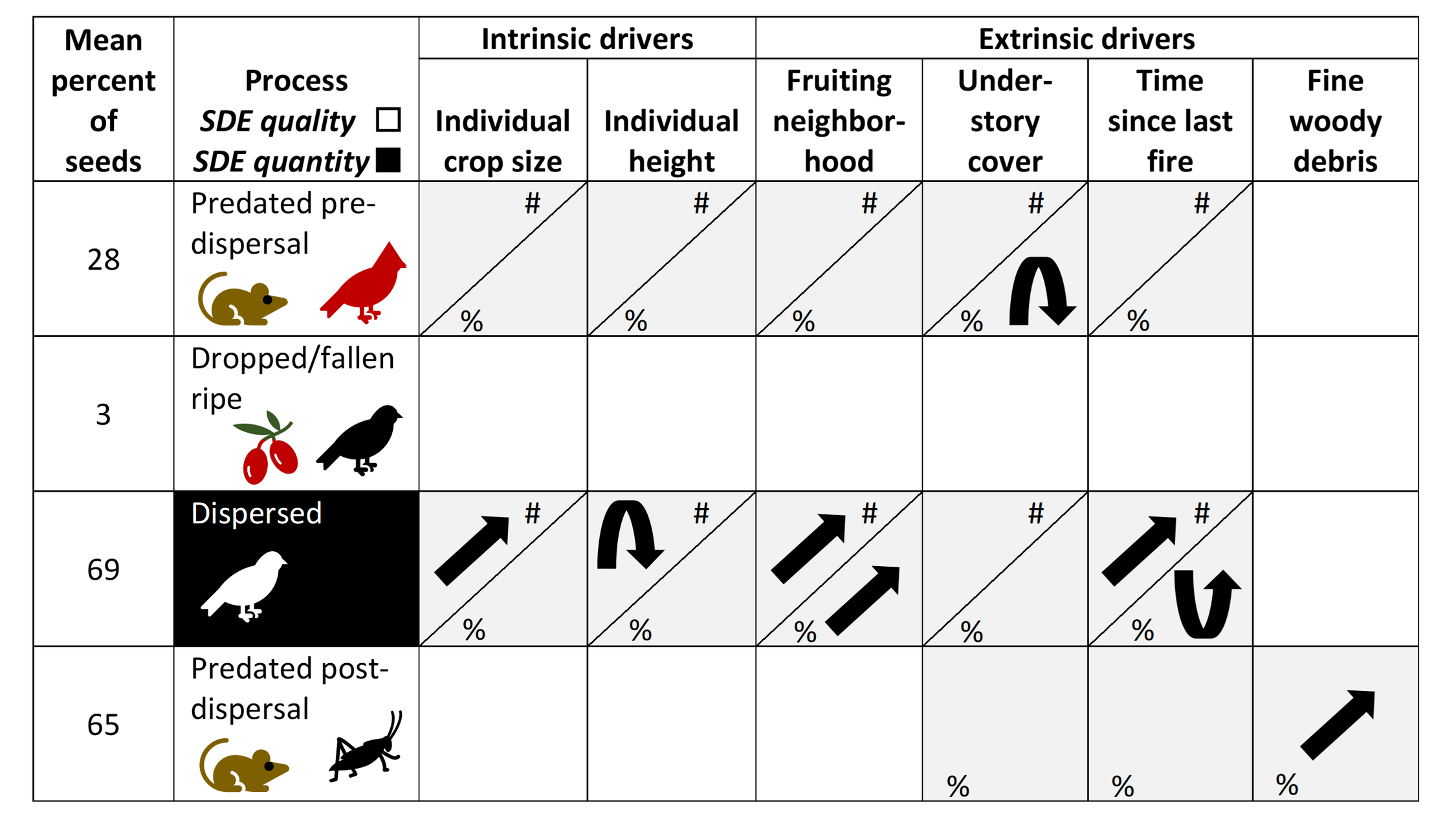

Supplement: S4 Fig — Cells shaded gray identify examined relationships between processes (second column rows) and drivers (remaining columns to the right). Straight arrows identify linear relationships and curvilinear arrows identify quadratic relationships (positive and negative) between the processes and drivers for both the numbers (#) and percentages (%) of seeds. Where no arrows are displayed, no significant relationships between the process and drivers were identified. (TIF) [file pone.0283810.s004.tif]
