# Supplementary material for: Multiple intrinsic and extrinsic drivers influence the quantity and quality components of seed dispersal effectiveness in the rare shrub Lindera subcoriacea
Source: PLoS One. 2023 Mar 31;18(3):e0283810. doi: 10.1371/journal.pone.0283810 (PMC10065295; doi:10.1371/journal.pone.0283810)
Supplement: S1 Table — (DOCX) [file pone.0283810.s005.docx]

|  | **Individual crop size** | **Individual height** | **5 m fruiting neighborhood** | **10 m fruiting neighborhood** | **30 m fruiting neighborhood** | **Understory cover** | **Time since last fire** |
| --- | --- | --- | --- | --- | --- | --- | --- |
| **Individual crop size** | 1.00 | 0.26 | -0.12 | -0.17 | 0.03 | 0.20 | -0.12 |
| **Individual height** | 0.26 | 1.00 | -0.20 | -0.21 | 0.31 | 0.43 | 0.23 |
| **5 m fruiting neighborhood** | -0.12 | -0.20 | 1.00 | 0.74 | 0.10 | 0.03 | 0.08 |
| **10 m fruiting neighborhood** | -0.17 | -0.21 | 0.74 | 1.00 | 0.10 | -0.04 | -0.12 |
| **30 m fruiting neighborhood** | 0.03 | 0.31 | 0.10 | 0.10 | 1.00 | 0.46 | -0.20 |
| **Understory cover** | 0.20 | 0.43 | 0.03 | -0.04 | 0.46 | 1.00 | -0.07 |
| **Time since last fire** | -0.12 | 0.23 | 0.08 | -0.12 | -0.20 | -0.07 | 1.00 |
