# Supplementary material for: Multiple intrinsic and extrinsic drivers influence the quantity and quality components of seed dispersal effectiveness in the rare shrub Lindera subcoriacea
Source: PLoS One. 2023 Mar 31;18(3):e0283810. doi: 10.1371/journal.pone.0283810 (PMC10065295; doi:10.1371/journal.pone.0283810)
Supplement: S2 Table — (DOCX) [file pone.0283810.s006.docx]

|  | **Understory cover** | **Time since last fire** | **%Litter** | **%Coarse woody debris** | **%Fine woody debris** | **%Bare ground** | **%Woody cover** | **%Herbaceous cover** |
| --- | --- | --- | --- | --- | --- | --- | --- | --- |
| **Understory cover** | 1.00 | 0.11 | -0.19 | 0.02 | 0.15 | 0.08 | -0.01 | -0.17 |
| **Time since last fire** | 0.11 | 1.00 | 0.17 | -0.07 | -0.04 | -0.25 | 0.12 | -0.20 |
| **%Litter** | -0.19 | 0.17 | 1.00 | 0.05 | 0.14 | -0.73 | 0.41 | -0.41 |
| **%Coarse woody debris** | 0.02 | -0.07 | 0.05 | 1.00 | 0.19 | -0.05 | -0.30 | -0.19 |
| **%Fine woody debris** | 0.15 | -0.04 | 0.14 | 0.19 | 1.00 | -0.08 | -0.07 | -0.27 |
| **%Bare ground** | 0.08 | -0.25 | -0.73 | -0.05 | -0.08 | 1.00 | -0.32 | 0.20 |
| **%Woody cover** | -0.01 | 0.12 | 0.41 | -0.30 | -0.07 | -0.32 | 1.00 | -0.26 |
| **%Herbaceous cover** | -0.17 | -0.20 | -0.42 | -0.19 | -0.27 | 0.20 | -0.26 | 1.00 |
