# Supplementary material for: Multiple intrinsic and extrinsic drivers influence the quantity and quality components of seed dispersal effectiveness in the rare shrub Lindera subcoriacea
Source: PLoS One. 2023 Mar 31;18(3):e0283810. doi: 10.1371/journal.pone.0283810 (PMC10065295; doi:10.1371/journal.pone.0283810)
Supplement: S3 Table — Confidence intervals that do not overlap with zero are significantly different. (DOCX) [file pone.0283810.s007.docx]

|  |  | **Confidence intervals** | |
| --- | --- | --- | --- |
| **Species pair** | **Mean difference** | **2.5 %** | **97.5 %** |
| American Robin–Northern Cardinal | -7 | -2.05 | -10.34 |
| American Robin–Red-headed Woodpecker | 12 | 8.13 | 16.27 |
| Northern Cardinal–Red-headed Woodpecker | 19 | 15.01 | 21.89 |
| Whited-eyed Vireo–American Robin | -22 | -18.69 | -25.68 |
| Whited-eyed Vireo–Northern Cardinal | -29 | -25.67 | -31.12 |
| Whited-eyed Vireo–Red-headed Woodpecker | -10 | -7.21 | -12.68 |
