# Supplementary material for: Multiple intrinsic and extrinsic drivers influence the quantity and quality components of seed dispersal effectiveness in the rare shrub Lindera subcoriacea
Source: PLoS One. 2023 Mar 31;18(3):e0283810. doi: 10.1371/journal.pone.0283810 (PMC10065295; doi:10.1371/journal.pone.0283810)
Supplement: S2 File — (DOCX) [file pone.0283810.s009.docx]

*Lindera subcoriacea* Seed Trap and Seed Depot Results

2022-06-29

# Seed trap results

## Understory effect on pre-dispersal predation (proportion)

First model (predation_understory.glmer) includes quadratic. predation_understory2.glmer does not include quadratic. predation_understory_null.glmer is null model

|  | npar | logLik | deviance | Chisq | Df | Pr(>Chisq) |
| --- | --- | --- | --- | --- | --- | --- |
| **Model without quadratic** | 3 | -137.5 | 275.1 | NA | NA | NA |
| **Model with quadratic** | 4 | -135.2 | 270.3 | 4.79 | 1 | 0.02862 |

|  | npar | logLik | deviance | Chisq | Df | Pr(>Chisq) |
| --- | --- | --- | --- | --- | --- | --- |
| **Null model** | 2 | -137.8 | 275.6 | NA | NA | NA |
| **Model without quadratic** | 4 | -135.2 | 270.3 | 5.271 | 2 | 0.0717 |

## Full model parameter estimates

| *Predictors* | *Log-Odds* | *std. Error* | *CI* | *p* |
| --- | --- | --- | --- | --- |
| (Intercept) | 0.08 | 0.66 | -1.22 – 1.38 | 0.904 |
| understory scale | 0.48 | 0.56 | -0.61 – 1.56 | 0.393 |
| understory scale^2 | -1.20 | 0.54 | -2.25 – -0.14 | **0.026** |
| N _ID_ | 42 | | | |
| Observations | 42 | | | |

## Individual crop size effect on dispersal (count)

|  | npar | logLik | deviance | Chisq | Df | Pr(>Chisq) |
| --- | --- | --- | --- | --- | --- | --- |
| **Null model** | 2 | -288.4 | 576.8 | NA | NA | NA |
| **Full model** | 3 | -239.6 | 479.1 | 97.74 | 1 | 4.763e-23 |

## Full model parameter estimates

| *Predictors* | *Log-Odds* | *std. Error* | *CI* | *p* |
| --- | --- | --- | --- | --- |
| (Intercept) | -1.34 | 0.48 | -2.28 – -0.41 | **0.005** |
| Ind seeds | 0.00 | 0.00 | -0.00 – 0.01 | 0.244 |
| N _ID_ | 42 | | | |
| Observations | 42 | | | |

## Stem height effect on dispersal (count)

|  | npar | logLik | deviance | Chisq | Df | Pr(>Chisq) |
| --- | --- | --- | --- | --- | --- | --- |
| **Model without quadratic** | 3 | -299.82 | 599.64 | NA | NA | NA |
| **Model with quadratic** | 4 | -283.73 | 567.46 | 32.178 | 1 | **<0.001** |

|  | npar | logLik | deviance | Chisq | Df | Pr(>Chisq) |
| --- | --- | --- | --- | --- | --- | --- |
| **Null model** | 2 | -308.35 | 616.70 | NA | NA | NA |
| **Model with quadratic** | 4 | 283.73 | 577.46 | 49.238 | 2 | **<0.001** |

## Full model parameter estimates

| *Predictors* | *Log-Odds* | *std. Error* | *CI* | *p* |
| --- | --- | --- | --- | --- |
| (Intercept) | 3.89 | 028 | 3.34 – 4.43 | **<0.001** |
| Ht scale | -0.16 | 0.13 | -0.42 – 0.10 | 0.238 |
| Ht scale^2 | 0.53 | 0.10 | -0.73 – -34 | **<0.001** |
| **Random Effects** | | | | |
| σ^2^ | 0.04 | | | |
| τ_00_ _Pop_Ind_ | 1.82 | | | |
| ICC | 0.98 | | | |
| N _Pop_Ind_ | 28 | | | |
| Observations | 42 | | | |
| Marginal R^2^ / Conditional R^2^ | 0.257 / 0.986 | | | |

## TSLB effects on dispersal (proportion)

|  | npar | logLik | deviance | Chisq | Df | Pr(>Chisq) |
| --- | --- | --- | --- | --- | --- | --- |
| **Model without quadratic** | 3 | -65.00 | 130.0 | NA | NA | NA |
| **Model with quadratic** | 4 | -58.03 | 116.1 | 13.95 | 1 | 0.0001877 |

|  | npar | logLik | deviance | Chisq | Df | Pr(>Chisq) |
| --- | --- | --- | --- | --- | --- | --- |
| **Null model** | 2 | -66.39 | 132.8 | NA | NA | NA |
| **Model with quadratic** | 4 | -58.03 | 116.1 | 16.73 | 2 | 0.0002326 |

## Full model parameter estimates

|  | **cbind(dispersed mod,Seeds mod-dispersed mod)** | | | |
| --- | --- | --- | --- | --- |
| *Predictors* | *Log-Odds* | *std. Error* | *CI* | *p* |
| (Intercept) | 3.01 | 0.50 | 2.02 – 4.00 | **<0.001** |
| tsb scale | -3.18 | 0.89 | -4.92 – -1.45 | **<0.001** |
| tsb scale^2 | 1.66 | 0.47 | 0.73 – 2.58 | **<0.001** |
| **Random Effects** | | | | |
| σ^2^ | 3.29 | | | |
| τ_00_ _Pop_Ind_ | 2.03 | | | |
| ICC | 0.38 | | | |
| N _Pop_Ind_ | 28 | | | |
| Observations | 42 | | | |
| Marginal R^2^ / Conditional R^2^ | 0.291 / 0.561 | | | |

## TSLB effects on dispersal (count)

First model (dispersalTot_tsb.glmer) includes quadratic. dispersalTot_tsb2.glmer does not include quadratic. dispersalTot_tsb_null.glmer is null model

|  | npar | logLik | deviance | Chisq | Df | Pr(>Chisq) |
| --- | --- | --- | --- | --- | --- | --- |
| **Model without quadratic** | 3 | -293.9 | 587.9 | NA | NA | NA |
| **Model with quadratic** | 4 | -293.9 | 587.9 | 0.006592 | 1 | 0.9353 |

|  | npar | logLik | deviance | Chisq | Df | Pr(>Chisq) |
| --- | --- | --- | --- | --- | --- | --- |
| **Null model** | 2 | -308.4 | 616.7 | NA | NA | NA |
| **Model without quadratic** | 3 | -293.9 | 587.9 | 28.81 | 1 | 7.996e-08 |

## Full model parameter estimates

|  | **dispersed mod** | | | |
| --- | --- | --- | --- | --- |
| *Predictors* | *Log-Mean* | *std. Error* | *CI* | *p* |
| (Intercept) | 3.41 | 0.30 | 2.81 – 4.00 | **<0.001** |
| tsb scale | 1.07 | 0.24 | 0.61 – 1.53 | **<0.001** |
| tsb scale^2 | -0.01 | 0.13 | -0.28 – 0.25 | 0.934 |
| **Random Effects** | | | | |
| σ^2^ | 0.04 | | | |
| τ_00_ _Pop_Ind_ | 2.13 | | | |
| ICC | 0.98 | | | |
| N _Pop_Ind_ | 28 | | | |
| Observations | 42 | | | |
| Marginal R^2^ / Conditional R^2^ | 0.339 / 0.989 | | | |

## Neighborhood effect on dispersal (proportion) 5 meters

|  | npar | logLik | deviance | Chisq | Df | Pr(>Chisq) |
| --- | --- | --- | --- | --- | --- | --- |
| **Model without quadratic** | 3 | -54.05 | 108.1 | NA | NA | NA |
| **Model with quadratic** | 4 | -53.46 | 106.9 | 1.179 | 1 | 0.2776 |

|  | npar | logLik | deviance | Chisq | Df | Pr(>Chisq) |
| --- | --- | --- | --- | --- | --- | --- |
| **Null model** | 2 | -57.36 | 114.7 | NA | NA | NA |
| **Model without quadratic** | 3 | -54.05 | 108.1 | 6.623 | 1 | 0.01007 |

## Full model parameter estimates

|  | **cbind(dispersed mod,Seeds mod-dispersed mod)** | | | |
| --- | --- | --- | --- | --- |
| *Predictors* | *Log-Odds* | *std. Error* | *CI* | *p* |
| (Intercept) | 4.63 | 0.63 | 3.40 – 5.86 | **<0.001** |
| NH seeds5 scale | 1.10 | 0.42 | 0.28 – 1.91 | **0.008** |
| NH seeds5 scale^2 | -0.47 | 0.42 | -1.29 – 0.35 | 0.263 |
| N _ID_ | 40 | | | |
| Observations | 40 | | | |

## Neighborhood effect on dispersal (proportion) 10 meters

|  | npar | logLik | deviance | Chisq | Df | Pr(>Chisq) |
| --- | --- | --- | --- | --- | --- | --- |
| **Model without quadratic** | 3 | -51.11 | 102.2 | NA | NA | NA |
| **Model with quadratic** | 4 | -50.57 | 101.1 | 1.085 | 1 | 0.2975 |

|  | npar | logLik | deviance | Chisq | Df | Pr(>Chisq) |
| --- | --- | --- | --- | --- | --- | --- |
| **Null model** | 2 | -57.36 | 114.7 | NA | NA | NA |
| **Model without quadratic** | 3 | -51.11 | 102.2 | 12.5 | 1 | 0.0004059 |

## Full model parameter estimates

|  | **cbind(dispersed mod,Seeds mod-dispersed mod)** | | | |
| --- | --- | --- | --- | --- |
| *Predictors* | *Log-Odds* | *std. Error* | *CI* | *p* |
| (Intercept) | 4.31 | 0.58 | 3.18 – 5.44 | **<0.001** |
| NH seeds10 scale | 1.47 | 0.48 | 0.53 – 2.40 | **0.002** |
| NH seeds10 scale^2 | 0.03 | 0.47 | -0.90 – 0.95 | 0.954 |
| N _ID_ | 40 | | | |
| Observations | 40 | | | |

## Neighborhood effect on dispersal (proportion) 30 meters

|  | npar | logLik | deviance | Chisq | Df | Pr(>Chisq) |
| --- | --- | --- | --- | --- | --- | --- |
| **Model without quadratic** | 3 | -48.48 | 96.96 | NA | NA | NA |
| **Model with quadratic** | 4 | -48.11 | 96.21 | 0.7457 | 1 | 0.3878 |

|  | npar | logLik | deviance | Chisq | Df | Pr(>Chisq) |
| --- | --- | --- | --- | --- | --- | --- |
| **Null model** | 2 | -52.91 | 105.81 | NA | NA | NA |
| **Model without quadratic** | 3 | -48.48 | 96.96 | 8.857 | 1 | 0.002919 |

## Full model parameter estimates

|  | **cbind(dispersed mod,Seeds mod-dispersed mod)** | | | |
| --- | --- | --- | --- | --- |
| *Predictors* | *Log-Odds* | *std. Error* | *CI* | *p* |
| (Intercept) | 3.84 | 0.52 | 2.82 – 4.86 | **<0.001** |
| NH seeds30 scale | 1.25 | 0.49 | 0.29 – 2.21 | **0.010** |
| NH seeds30 scale^2 | 0.06 | 0.49 | -0.90 – 1.01 | 0.907 |
| N _ID_ | 34 | | | |
| Observations | 34 | | | |

## Neighborhood effect on dispersal (count) 5 meters

|  | npar | logLik | deviance | Chisq | Df | Pr(>Chisq) |
| --- | --- | --- | --- | --- | --- | --- |
| **Model without quadratic** | 3 | -175 | 350.0 | NA | NA | NA |
| **Model with quadratic** | 4 | -174 | 348.1 | 1.948 | 1 | 0.1628 |

|  | npar | logLik | deviance | Chisq | Df | Pr(>Chisq) |
| --- | --- | --- | --- | --- | --- | --- |
| **Null model** | 2 | -178 | 355.9 | NA | NA | NA |
| **Model without quadratic** | 3 | -175 | 350.0 | 5.909 | 1 | 0.01507 |

## Full model parameter estimates

|  | **dispersed mod** | | | |
| --- | --- | --- | --- | --- |
| *Predictors* | *Log-Mean* | *std. Error* | *CI* | *p* |
| (Intercept) | 3.33 | 0.19 | 2.95 – 3.70 | **<0.001** |
| NH seeds5 scale | 0.42 | 0.15 | 0.13 – 0.71 | **0.004** |
| NH seeds5 scale^2 | -0.20 | 0.15 | -0.49 – 0.09 | 0.168 |
| N _ID_ | 40 | | | |
| Observations | 40 | | | |

## Neighborhood effect on dispersal (count) 10 meters

|  | npar | logLik | deviance | Chisq | Df | Pr(>Chisq) |
| --- | --- | --- | --- | --- | --- | --- |
| **Model without quadratic** | 3 | -175.7 | 351.4 | NA | NA | NA |
| **Model with quadratic** | 4 | -173.9 | 347.9 | 3.549 | 1 | 0.05958 |

|  | npar | logLik | deviance | Chisq | Df | Pr(>Chisq) |
| --- | --- | --- | --- | --- | --- | --- |
| **Null model** | 2 | -178.0 | 355.9 | NA | NA | NA |
| **Model without quadratic** | 3 | -175.7 | 351.4 | 4.523 | 1 | 0.03345 |

## Full model parameter estimates

|  | **dispersed mod** | | | |
| --- | --- | --- | --- | --- |
| *Predictors* | *Log-Mean* | *std. Error* | *CI* | *p* |
| (Intercept) | 3.41 | 0.20 | 3.03 – 3.80 | **<0.001** |
| NH seeds10 scale | 0.38 | 0.14 | 0.10 – 0.65 | **0.007** |
| NH seeds10 scale^2 | -0.29 | 0.15 | -0.59 – 0.01 | 0.060 |
| N _ID_ | 40 | | | |
| Observations | 40 | | | |

# Seed Depot results

## Fine woody debris effect on post-dispersal predation (proportion)

| Analysis of Deviance Table | | | | |
| --- | --- | --- | --- | --- |
| **Resid. Df** | **Resid. Dev** | **Df** | **Deviance** | **Pr(>Chi)** |
| 54 | 171.3 | NA | NA | NA |
| 55 | 175.2 | -1 | -3.914 | 0.04789 |
